# Supplementary material for: A mechanical modeling framework to study endothelial permeability
Source: Biophys J. 2024 Jan 1;123(3):334–48. doi: 10.1016/j.bpj.2023.12.026 (PMC10870174; doi:10.1016/j.bpj.2023.12.026)
Supplement: Document S1. Figures S1–S11 and Table S1 [file mmc1.pdf]

Biophysical Journal, Volume *123*

Supplemental information

A mechanical modeling framework to study endothelial permeability

Pradeep Keshavanarayana and Fabian Spill

S1 Source files

Link to download all the source files:
https://github.com/bkprdp/VE-Cadherin-Mechanical-Model-Abaqus
File descriptions :

1. `Cell_UMAT_Small_Strain.f`: User-defined material. Active and Passive stress growth, using small strain formulation.
2. `UAMP` : Random pressure amplitude
3. `Planar_Monolayer_Without_ECM.inp` : inp file for planar monolayer without ECM
4. `Planar_Monolayer_With_ECM.inp` : inp file for planar monolayer with ECM
5. `Cylindrical_Monolayer_Without_ECM.inp` : inp file for cylindrical monolayer without ECM
6. `Cylindrical_Monolayer_With_Rigid_ECM.inp` : inp file for cylindrical monolayer with fixed radial constraint representing rigid ECM
7. `Nodes_Bi_Tri_Cellular_Jncs.py` : Python script to classify nodes belonging to bi-cellular and tri-cellular junctions
8. `Planar_Monolayer_COPEN_Analysis.py` : Python script to perform quantitative analysis of the csv file containing copen values

Animations of the simulations are added as supplemental material.

S2 Traction-Separation law

In this article, traction-separation law is hypothesised to be the mechanical equivalent of the catch-slip bond law. The parameters of traction-separation law could be varied to modify the association and dissociation behaviour of VE-cadherin bonds. As explained in the main text, only three parameters are needed :

1. Stiffness of VE-cadherin bond
2. Stretch at which the VE-cadherin bond starts to dissociate
3. Stretch at which the VE-cadherin bond is completely dissociated

It can be seen in Figure S1, as the normalised stiffness is increased from 1 to 2, the maximum contact force that the bond can take is also doubled. When the maximum separation that the bond can handle before complete dissociation is increased, the time the bond takes to dissociate also increases. It can be easily seen that by changing δ_i^0, stiffness can be changed as well. Thus, by varying these parameters, quantitative comparison with experiments is possible.

S3 Cauchy stress tensor derivation

Any unit vector $\mathbf{m}$ along the fibre direction (ω,ϕ), Fig. S2, can be written as

$$\mathbf{m} = sin(\omega)cos(\phi)\mathbf{x_1} + sin(\omega)sin(\phi)\mathbf{x_2} + cos(\omega)\mathbf{x_3} \tag{1}$$

Following [1], we can write the components of active Cauchy stress tensor as

$$\sigma^a_{ij} = \frac{3}{4\pi}\int_0^{2\pi}\int_0^{\pi}\sigma^a(\omega,\phi)m_i m_j sin(\omega)d\omega d\phi \tag{2}$$

where i,j = 1,2,3. In the case of 2D geometry,

$$\sigma^a_{ij} = \frac{1}{\pi}\int_{-\pi/2}^{\pi/2}\sigma^a(\phi)m_i m_j d\phi \tag{3}$$

where i,j=1,2.

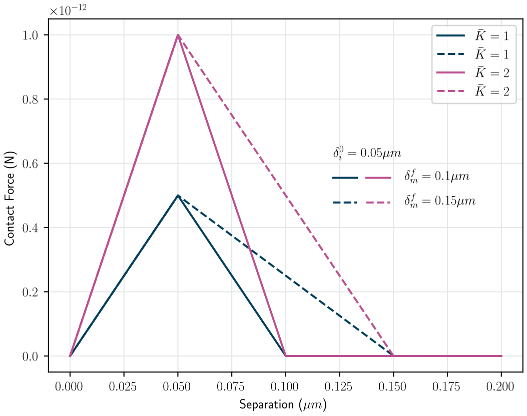

Figure S1: Parameters involved in traction separation law can be varied to study the maximum force, time of association and dissociation of VE-cadherin bonds. These parameters could also be used for simulating the differences in cell-cell and cell-ECM bonds.

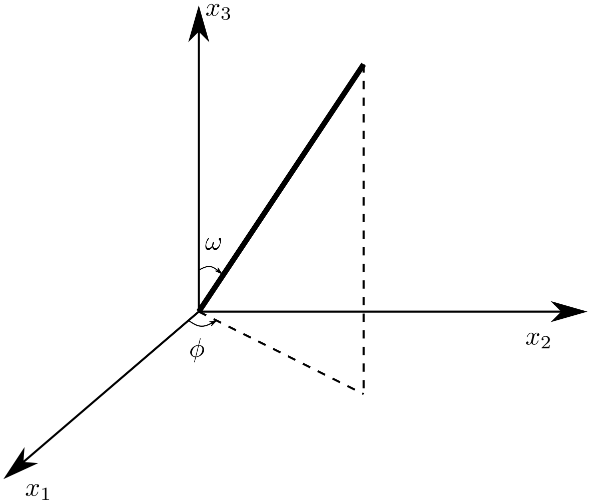

Figure S2: Orientation of fibre with respect to x_1, x_2, and x_3 axes. ω is the angle between the fibre and x_3 axis, while ϕ is the angle between the projection of fibre on x_1-x_2 plane and the x_1 axis.

S4 Finite Strain formulation

In this article, all the simulations presented used small strain formulation. This can be further extended to finite strain formulation using non-linear hyper-elastic materials. Here an example with Neo-Hookean material is given. Passive stress can thus be formulated with the strain energy function

$$\psi(I_1, J) = C_{10}(I_1 - 3) + \frac{1}{D_1}(J-1)^2 \tag{4}$$

where ψ is the strain energy density function, I_1 is the first invariant of the left Cauchy-Green tensor $\mathbf{B}$, J is the determinant of the deformation gradient $\mathbf{F}$. C_{10} and D_1 are the material parameters related to Young's modulus and Poisson ratio via Bulk modulus (K_0) and shear modulus (μ_0) as given in Eq. (5).

$$\begin{aligned} C_{10} &= \frac{\mu_0}{2} \\ D_1 &= \frac{2}{K_0} \end{aligned} \tag{5}$$

Left Cauchy-Green tensor is defined as

$$\mathbf{B} = \mathbf{F}.\mathbf{F}^{\mathbf{T}} \tag{6}$$

The constitutive equation is given by the first derivative of the strain energy function

$$\sigma_{ij}^p = \frac{2}{J}C_{10}\left(\bar{B}_{ij} - \frac{1}{3}\delta_{ij}\bar{B}_{kk}\right) + \frac{2}{D_1}(J-1)\delta_{ij} \tag{7}$$

where δ_{ij} is the Kronecker delta, and $i, j = 1, 2, 3$. $\bar{\mathbf{B}}$ is the volumetric strain tensor given by

$$\bar{\mathbf{B}} = J^{\left(\frac{-2}{3}\right)}\mathbf{B} \tag{8}$$

In general, the Jacobian matrix $\mathbf{C}$ needed for the Newton scheme used by ABAQUS, can be obtained from the equilibrium equation

$$\delta\boldsymbol{\tau} = J\mathbf{C} : \delta\mathbf{D} \tag{9}$$

where $\boldsymbol{\tau}$ is the Kirchoff stress, related to the Cauchy stress as $J\boldsymbol{\sigma}$. The rate of deformation $\mathbf{D}$ is defined as

$$\delta\mathbf{D} = \mathbf{sym}\left(\delta\mathbf{F}.\mathbf{F^{-1}}\right) \tag{10}$$

Solution of Eq. 9 can be obtained numerically by perturbing the individual component of deformation gradient $\mathbf{F}$ by a small amount ϵ as described in [2, 3]. Derivation leads to

$$\mathbf{C} = \frac{1}{J\varepsilon}\left[\boldsymbol{\tau}\left(\hat{\mathbf{F}}\right) - \boldsymbol{\tau}\left(\mathbf{F}\right)\right] \tag{11}$$

where $\hat{\mathbf{F}}$ is the perturbed deformation gradient given as

$$\hat{F}_{ij} = F_{ij} + \Delta F_{ij} \tag{12}$$

In addition, in the case of finite strain formulation, the rotation of fibres has to be taken into account during the evaluation of the active stress tensor. Thus (2) becomes

$$\sigma^a_{ij} = \frac{3}{4\pi}\int_0^{2\pi}\int_0^{\pi} \sigma^a(\omega,\phi) m^*_i m^*_j sin(\omega) d\omega d\phi \tag{13}$$

where m^*_i, m^*_j are the vectors in the current configuration evaluated as

$$m^*_i = \mathbf{R}m_i \tag{14}$$

where $\mathbf{R}$ is the rotation matrix evaluated via the decomposition of the deformation gradient as

$$\mathbf{F} = \mathbf{RU} \tag{15}$$

S5 Parameter values

Parameter values used in this article are given in Tab. S1. Some of the parameter values are obtained directly from the literature while some of them are modified to obtain qualitatively comparable results with the experiments.

Parameters	Value	Units	Reference
Passive stiffness	10	kPa	[4]
Poisson ratio	0.45	-	-
Max stress in stress fibre	1	MPa	[5]
Strain rate coefficient	0.1	s	[5]
k_f	0.01	/s	[5]
k_b	0.1	/s	[5]
Bond stiffness	1	N/m	[6]
Damage initiation	0.05	μm	[6]
Damage termination	0.15	μm	[6]
Cadherin Concentration	1e7	cadherins/m^2	-

Table S1: Baseline parameter values

S6 Convergence study

Mesh convergence analysis is performed. It can be seen in Fig. S3 that the error (evaluated wrt to results from a very fine mesh simulation) with a mesh size of 1μm is 5%. We assume that this error is acceptable for the qualitative nature of this article. Hence all the analyses performed in this article were performed with the mesh with an element size of 1μm.

S7 Effect of $\hat{C}$ on permeability

As discussed in the article, active stress, evaluated based on the calcium concentration $\hat{C}$ has a direct influence on the permeability. When $\hat{C}$ increases, the active stress in the cytoplasm increases resulting in increased permeability as shown in Fig. S4. Since an increase in cytoplasmic calcium concentration further increases the tension on cell-cell junctions, as explained in the article, intercellular random pressure load factor $\hat{\zeta}_p$ is assumed to increase with $\hat{C}$. This relation is assumed to behave as shown in Fig. S5. Further, we also show how the permeability varies with time, Fig. S7. We also saw that when ζ_p increases along with $\hat{C}$, the increase in permeability is higher than the case where ζ_p is held constant (Fig. S4). Hence, in all the simulations presented in the article, we vary ζ_p along with $\hat{C}$ following Fig. S5. The relation between ECM stiffness E and $\hat{C}$ used in this article is shown in Fig. S6.

S8 Uniform vs Random gap opening

A comparison of gap opening, when the contraction is uniform and random, is shown in Fig. S8. The simulated animations of the deformation of the endothelial monolayer, in case of uniform and random loading, can be found in the link provided in supplemental section S1

S9 Disturbed flow vs uniform flow

We define permeability as the ratio of the number of open junctions to the total number of junctions in the monolayer. As expected, when the shear stress gradient between cells increases, the probability of cell-cell junctions being open increases. In this regard, we saw that in disturbed flow conditions, where the flow is randomly varying in both x and y directions, permeability was slightly higher than that of a uniform flow condition, where all cells experience the same flow, as shown in Fig. S9. It is to be noted that the current analysis shows that the difference between uniform and disturbed flow is very small. This might be either due to the values of parameters chosen in this study or due to the lack of specific mechano-sensory channels that can sense flow such as glycocalyx. Thus, in future, this analysis will be coupled with glycocalyx which is found to play an important role in regulating permeability as well [7]. Further development could help us understand the role of flow in diseases such as atherosclerosis and other permeability-related diseases.

S10 Thickness of cell

In the article, we consider the thickness of the cell as 0.1μm. However, in literature, it has been found that thickness varies to 10 μm. In this regard, we varied the thickness of the cell and studied its effect on permeability. As the thickness of the cell is increased, keeping the cadherin concentration constant, the net force that cell-cell contact can resist without damage increases. But simultaneously, the contractile force and force due to random pressure loading on the boundary also increase. Hence the contributions balance out and we do not observe any difference in permeability due to the changing thickness of the cell as can be seen in Fig. S10. In future, this study could be extended by considering the effect of the nucleus, which will alter cell stiffness locally.

S11 Size of monolayer

We study the effect of the number of cells in the monolayer on permeability. We found that with a low number of cells in the monolayer, the variations in the magnitude of permeability at tri-cellular junctions were very high compared to that of the monolayer with 42 cells, as seen in Fig. S11b, Fig. S11c. This is due to the increased number of tri-cellular junctions used for evaluating the average permeability. In addition, as the number of cells increased, the difference in permeability between the endothelial monolayers also decreased, as shown in Fig. S11d. So, we have used a monolayer with 32 cells in all the simulations.

S12 Pseudocode

The pseudocode describing the methodology followed in the analysis presented in the article is given below.

```
Define geometry and material properties
Initial conditions (u = 0, ϵ = 0, ϵ̇ = 0, η = 0, Σ = 0)
Contact definitions
while t ≤ t_end do                                              ▷ For each time step
    while N ≤ N_ele do                                          ▷ For each element
        function EVALUATE TOTAL STRESS(u,ϵ,Σ)
            EVALUATEACTIVESTRESS(u,ϵ̇,η,σ^a)
            EVALUATEPASSIVESTRESS(u,ϵ,σ^p)
            return Σ ← σ^a + σ^p
        end function
        function EVALUATE LOAD AND BOUNDARY CONDITIONS
            if ECM is considered then
                Evaluate Cell-ECM contact
                Evaluate Cell-Cell contact
            else if ECM is not considered then
                Evaluate Cell-Cell contact
            end if
            APPLY INTERCELLULAR RANDOM PRESSURE                 ▷ Applied at cell-cell interface
            if True then
                APPLY INTERCELLULAR SHEAR STRESS                ▷ Applied at cell-cell interface
            end if
            if True then
                APPLY TRACTION DUE TO DISTURBED FLOW            ▷ Applied on top of cells
            end if
        end function
    end while
    STATICANALYSIS(Termination Criteria)
    Update (u, ϵ, ϵ̇, η)
end while
POST PROCESSING TO EVALUATE PERMEABILITY
```

S13 Simulating a specific experiment

The modelling framework presented in this article is a general computational methodology where the principles of contact mechanics have been used to study endothelial permeability. The model can be applied successfully to study a wide range of experiments either by changing the material parameters or the relation between the variables present in the model. The data-driven modifications to the model could give deeper insights into the specificities of the experiment at hand. The changes that could be made to the model with the help of data obtained from the experiments are

1. Geometry of the cell and the monolayer
2. Material parameters of the cell and the substrate
3. Material parameters for cell-cell junctions and cell-substrate junction
4. Relation between calcium concentration and intercellular pressure
5. Relation between ECM stiffness and calcium concentration
6. Distribution of random forcing functions
7. Exact distribution of force due to disturbed flow on endothelial cells evaluated using FSI or other experimental methods.

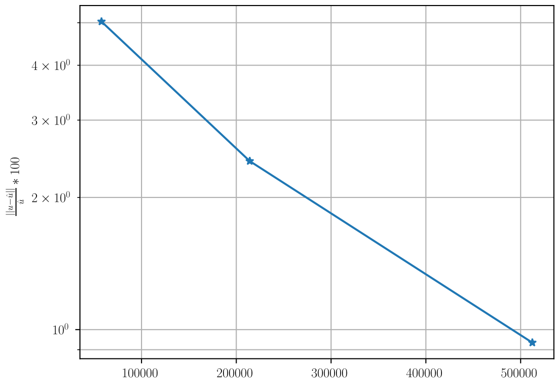

Figure S3: Mesh convergence study. Error for mesh size of 1μm is less than 5%. Hence all simulations use a mesh size of 1μm.

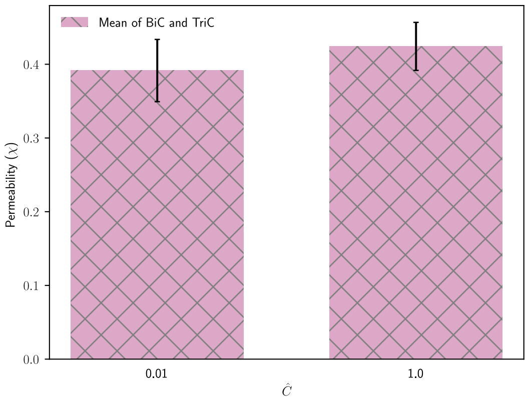

Figure S4: Increase in permeability due to increase in cytoplasmic calcium concentration keeping the intercellular pressure load factor constant. ($\hat{\zeta}_p = 0.01$ MPa). The increase is only due to an increase in active stress.

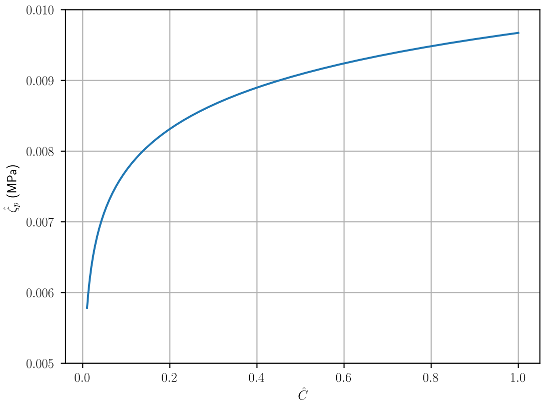

Figure S5: Relation between intercellular random pressure load factor $\hat{\zeta}_p$ and cytoplasmic calcium concentration $\hat{C}$.

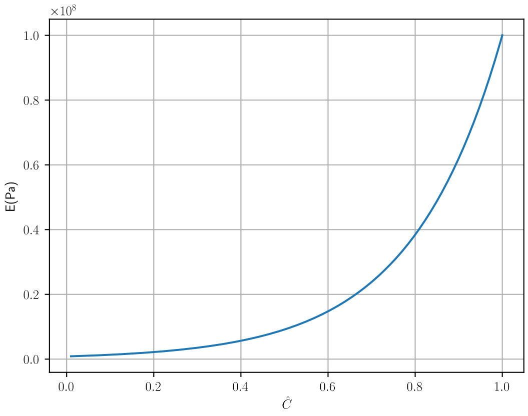

Figure S6: Relation between ECM stiffness and cytoplasmic calcium concentration $\hat{C}$.

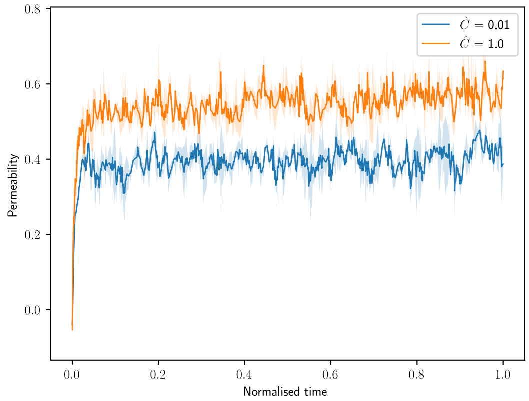

Figure S7: Variation of permeability over time. With an increase in $\hat{C}$, permeability increases.

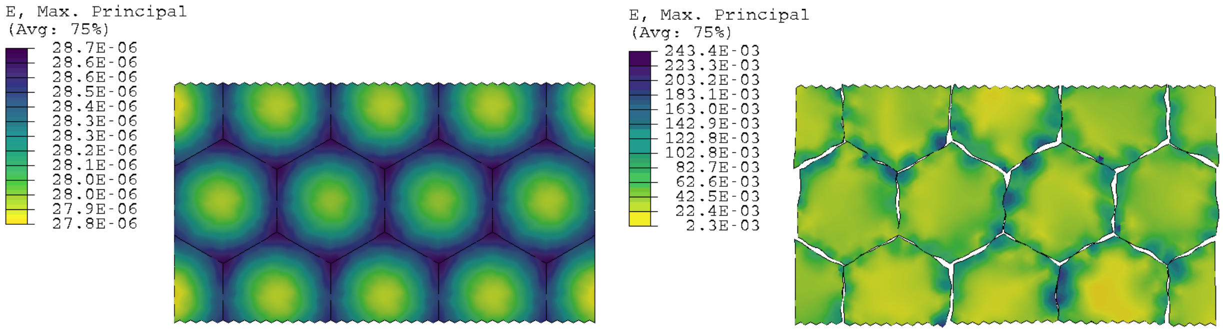

(a) (b)

Figure S8: Strain field in a planar monolayer subjected to different types of loading. a) Due to the uniform contraction of cells, strain is high at a tri-cellular junction compared to the bi-cellular junction. b) Due to random loads between cells, strain is high either at a bi-cellular or tri-cellular junction depending on the load. Thus, the location of high permeability depends on the strain distribution in the endothelial monolayer.

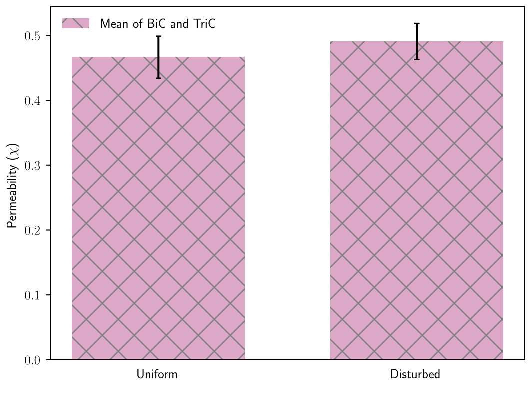

Figure S9: Variation in permeability due to uniform and disturbed flows. The difference in permeability is not high. This indicates the need for the inclusion of suitable mechano-sensors such as glycocalyx in the model.

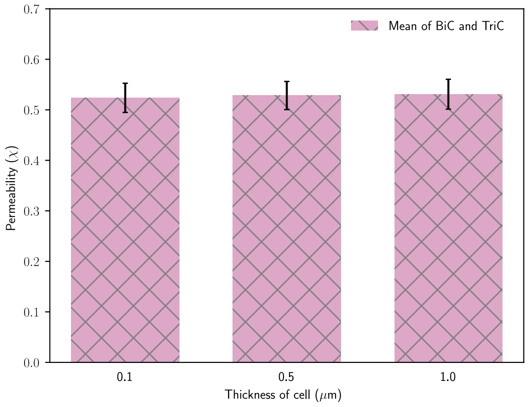

Figure S10: Variation in permeability due to change in thickness of the cell. The difference in permeability due to changing thickness is not significant.

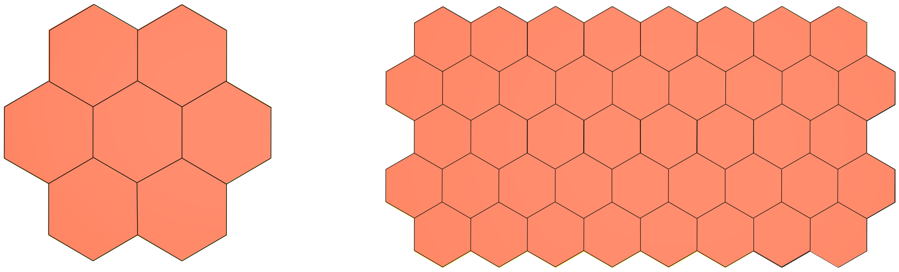

(a)

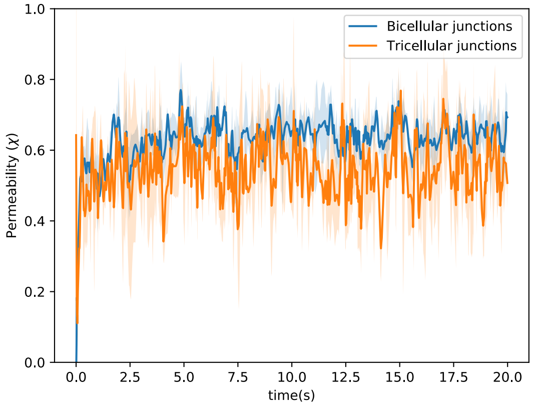

(b)

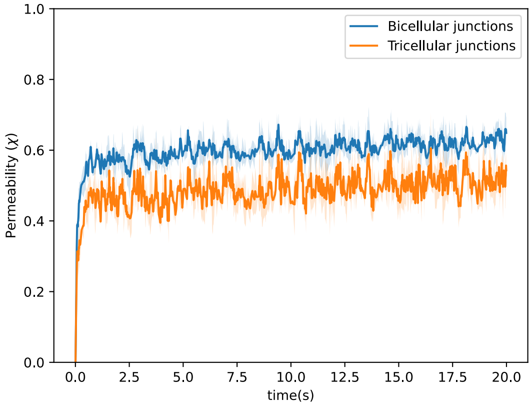

(c)

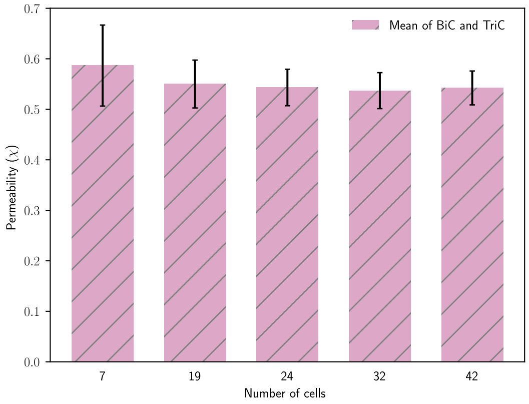

(d)

Figure S11: Variation in permeability over time for planar monolayer with differing number of cells. a) Schematic of planar monolayer with 7 and 42 cells. b) The variation in permeability at tri-cellular junctions in the case of the monolayer with 7 cells is very high. This is because there are only 6 tricellular junctions available over which the average is evaluated. c) Upon an increase in the number of tri-cellular junctions by increasing the number of cells to 42, the behaviour is smoothened. d) Upon an increase in the number of cells in the monolayer from 7 to 19, we observe a drop in the permeability. However, upon increasing the number of cells further, permeability does not vary much. We use 32 cells in all the simulations using the planar endothelial monolayer in this article.

Bibliography

[1] V. S. Deshpande, R. M. McMeeking, and A. G. Evans, "A model for the contractility of the cytoskeleton including the effects of stress-fibre formation and dissociation," *Proc. R. Soc. A Math. Phys. Eng. Sci.*, vol. 463, no. 2079, pp. 787–815, 2007.

[2] C. Miehe, "Numerical computation of algorithmic (consistent) tangent moduli in large-strain computational inelasticity," *Comput. Methods Appl. Mech. Eng.*, vol. 134, no. 3-4, pp. 223–240, 1996.

[3] Wei Sun, Elliot L. Chaikof, and M. E. Levenston, "Numerical Approximation of Tangent Moduli for Finite Element Implementations of Nonlinear Hyperelastic Material Models," *J Biomech Eng.*, vol. 130, no. 6, 2008.

[4] H. Sato, M. Katano, T. Takigawa, and T. Masuda, "Estimation for the elasticity of vascular endothelial cells on the basis of atomic force microscopy and Young's modulus of gelatin gels," *Polym. Bull.*, vol. 47, no. 3-4, pp. 375–381, 2001.

[5] P. Keshavanarayana, M. Ruess, and R. de Borst, "A feedback-loop extended stress fiber growth model with focal adhesion formation," *Int. J. Solids Struct.*, vol. 128, pp. 160–173, 2017.

[6] P. Panorchan, J. P. George, and D. Wirtz, "Probing Intercellular Interactions between Vascular Endothelial Cadherin Pairs at Single-molecule Resolution and in Living Cells," *J. Mol. Biol.*, vol. 358, no. 3, pp. 665–674, 2006.

[7] R. O. Dull and R. G. Hahn, "The glycocalyx as a permeability barrier: basic science and clinical evidence," *Crit. Care*, vol. 26, no. 1, pp. 1–8, 2022.
