# Supplementary material for: A mechanical modeling framework to study endothelial permeability
Source: Biophys J. 2024 Jan 1;123(3):334–48. doi: 10.1016/j.bpj.2023.12.026 (PMC10870174; doi:10.1016/j.bpj.2023.12.026)
Supplement: Document S2. Article plus supporting material [file mmc7.pdf]

Biophysical Journal
Article

A mechanical modeling framework to study endothelial permeability

Pradeep Keshavanarayana[1,*] and Fabian Spill[1,*]
[1]School of Mathematics, University of Birmingham, Birmingham, United Kingdom

ABSTRACT The inner lining of blood vessels, the endothelium, is made up of endothelial cells. Vascular endothelial (VE)-cadherin protein forms a bond with VE-cadherin from neighboring cells to determine the size of gaps between the cells and thereby regulate the size of particles that can cross the endothelium. Chemical cues such as thrombin, along with mechanical properties of the cell and extracellular matrix are known to affect the permeability of endothelial cells. Abnormal permeability is found in patients suffering from diseases including cardiovascular diseases, cancer, and COVID-19. Even though some of the regulatory mechanisms affecting endothelial permeability are well studied, details of how several mechanical and chemical stimuli acting simultaneously affect endothelial permeability are not yet understood. In this article, we present a continuum-level mechanical modeling framework to study the highly dynamic nature of the VE-cadherin bonds. Taking inspiration from the catch-slip behavior that VE-cadherin complexes are known to exhibit, we model the VE-cadherin homophilic bond as cohesive contact with damage following a traction-separation law. We explicitly model the actin cytoskeleton and substrate to study their role in permeability. Our studies show that mechanochemical coupling is necessary to simulate the influence of the mechanical properties of the substrate on permeability. Simulations show that shear between cells is responsible for the variation in permeability between bicellular and tricellular junctions, explaining the phenotypic differences observed in experiments. An increase in the magnitude of traction force due to disturbed flow that endothelial cells experience results in increased permeability, and it is found that the effect is higher on stiffer extracellular matrix. Finally, we show that the cylindrical monolayer exhibits higher permeability than the planar monolayer under unconstrained cases. Thus, we present a contact mechanics-based mechanochemical model to investigate the variation in the permeability of endothelial monolayer due to multiple loads acting simultaneously.

SIGNIFICANCE A novel mechanical modeling framework is introduced to study the role of mechanochemical stimuli in regulating the gaps between endothelial cells. The model uses the principles of mechanics to simulate the bond formed between adhesion proteins on neighboring cells, which are responsible for maintaining cell-cell contact. Our simulations showed that gaps between cells are controlled not only by cell-cell interactions but also by cell-substrate interactions. Our model predicts that mechanochemical coupling between matrix stiffness and calcium signaling is driving permeability. The mechanochemical modeling framework provides the right setting to study the effect of mechanical and chemical stimuli acting simultaneously on endothelial monolayers of varying geometrical and mechanical properties.

INTRODUCTION

The vascular network is responsible for the transport of blood and other essential nutrients across the body via arteries, veins, and capillaries. Arteries carry the blood away from the heart, veins bring the blood back to the heart while capillaries act as a bridge between arteries and veins, and ensure the delivery of blood, nutrients, and oxygen to the surrounding tissue. The blood vessels are made up of a specialized type of cells called vascular endothelial (VE) cells. They form a network of cylindrical monolayers, termed the endothelium, allowing fluid to flow within (1). In the case of arteries and veins, the endothelium is surrounded by smooth muscle tissues while capillaries are made up of a single layer of endothelial cells (2). Capillaries are part of the microcirculation and are the smallest blood vessels in the human body (3).

In a healthy vascular system, the homeostasis of capillaries is implied by a well-regulated bidirectional transport of material between the endothelium and the surrounding tissues. The transport can occur via two pathways: transcellular (through the cell) and paracellular (between the cells)

Submitted August 9, 2023, and accepted for publication December 28, 2023.
*Correspondence: p.keshavanarayana@bham.ac.uk or f.spill@bham.ac.uk
Editor: Guy Genin.
https://doi.org/10.1016/j.bpj.2023.12.026
© 2023 Biophysical Society.
This is an open access article under the CC BY license (http://creativecommons.org/licenses/by/4.0/).

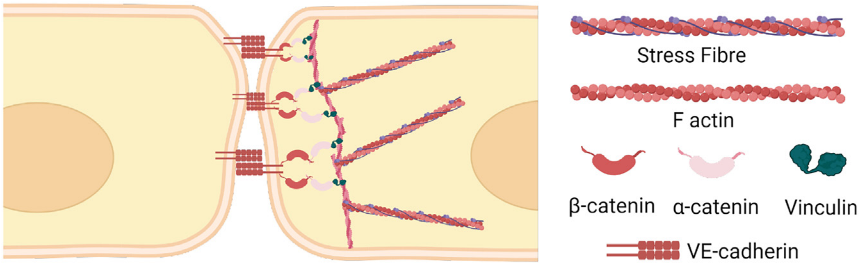

FIGURE 1 Schematic of proteins involved in cell-cell junction mechanotransduction. VE-cadherins from neighboring cells form a homophilic bond as part of the adherens junctions. VE-cadherins are attached to the catenin family of proteins, which, under tension, are connected to the cytoskeleton via vinculin. Created with biorender.com. To see this figure in color, go online.

(2). Transcellular transport involves endocytosis (into the cell) and exocytosis (out of the cell). These processes are highly selective and it is found that only a few micronutrients such as vitamin B12 follow the transcellular pathway (4). On the other hand, the paracellular pathway is regulated by several intercellular junctional proteins that regulate the gap size between the cells. Most of the materials passing the endothelium follow the paracellular pathway and they have to overcome the barrier properties of these interjunctional proteins (2). One of the intercellular junctions is termed the adherens junction and is commonly found in the vascular endothelium. VE-cadherin proteins are the major component of adherens junctions. They form a homophilic bond with VE-cadherin proteins on the neighboring cells and dictate the size of material that can pass the endothelium (5). It is to be noted that the VE-cadherin-VE-cadherin homophilic bond is highly dynamic in nature, resulting in the constant opening and closing of gaps due to changes in cellular and extracellular properties (6). The number and size of gaps present decide the amount of material that can successfully cross the endothelium, which is commonly termed (vascular) permeability. Thus, an increase in the amount of material that can overcome the endothelial barrier indicates higher endothelial permeability. Along with VE-cadherin-based adherens junctions, claudin and occludin proteins form tight junctions regulating vascular permeability. Since tight junctions are mainly found in specialized microvasculature such as the blood-brain barrier (2), we do not consider them in this study.

Dysregulation of VE-cadherin homophilic bonds may lead to either larger gaps or changes in the frequency of gap formation or gaps not being formed, leading to abnormal permeability. Some cases of diseased states where abnormal permeability is found are cancer (7), atherosclerosis (8), asthma (9), and COVID-19 (10). Cancer metastasis, a key characteristic of cancer progression, involves the spread of cancer cells from their origin to other body parts via the vascular network. This metastasis is facilitated by abnormal vascular permeability, which allows for intravasation (entry into blood vessels) and extravasation (exit from blood vessels) of cancer cells (7). Similarly, abnormal permeability plays a significant role in atherosclerosis, a prevalent cardiovascular disease in humans. This condition is characterized by the infiltration of fat and cholesterol molecules from the blood through the endothelial layer, subsequently accumulating on the surrounding membrane (8). This accumulation narrows the blood vessels, disrupting normal blood flow, and, in severe cases, may result in cardiac arrest (11). Vascular permeability has a major role in both acute and chronic inflammation since leukocytes have to cross the endothelial barrier and reach the tissues (2). VE-cadherin-based cell-cell junctions are also found to play an important role in angiogenesis and vascular morphogenesis (12,13). Thus, it becomes prudent to understand the physiology of variation of permeability of the endothelium. A better knowledge of how VE-cadherin bonds could be regulated helps us to understand the disease pathology and develop cures.

VE-cadherins are connected downstream to the actin cytoskeleton via the catenin and vinculin family of proteins (14), as shown in Fig. 1. Thus, regulation of the actin cytoskeleton is expected to affect the binding properties of the VE-cadherins also. Experiments have shown how modifying the contractility of actin-myosin stress fibers via Rho/ROCK pathways affects permeability (15). Chemical cues such as thrombin increase permeability (16) while S1P stabilizes the cell-cell junctions and reduces permeability (17). In addition to chemical cues, mechanical cues are also found to affect cell-cell junctions. Properties of the microenvironment such as stiffness of the substrate (18), geometric properties of the substrate (19), and mechanical stimuli such as cyclic stretch (20) are all known to affect permeability. But in vivo, chemical and mechanical stimuli act simultaneously and there is a coupling between them. Experiments carried out in vitro usually study either the effect of chemical or mechanical stimuli individually. In this regard, mathematical models that can integrate different mechanochemical features of the cell play an important role in bridging the understanding between individual responses observed experimentally.

Over the past few decades, several mathematical models have been developed to understand the behavior of endothelial cells in differing mechanical and chemical microenvironments. A mathematical model consisting of a system

of reaction-diffusion equations along with reinforced random walk governing endothelial cell migration predicted the pattern formation of endothelial cells on biogel surfaces (21). The model found that steady patterns of hexagonal endothelial cells could be formed only when the diffusion of the growth factor was low and its rate of decay was high. However, this model was unable to incorporate remodeling of the pattern of endothelial cells as a response to the continuously changing microenvironment. To study effects such as directed motility of endothelial cells, a 2D cellular Potts-based model was developed with positive feedback between cell displacement and polarity (22). The model was used to study the statistical properties of the random streaming behavior of the endothelial monolayer and predicted that, in monolayer cultures, both the speed and persistence of cell motion decrease compared with that of single cells. While the high-level active response of endothelial monolayers could be studied using such feedback loops, studying the effect of low-level gene data on high-level migration was not possible. A dynamic cell-steering multiscale model was thus developed that studied the effect of gene-level data on the migration of endothelial cells (23). The model provided a mechanistic explanation for the genetic modularity observed during the dynamic reorganization of endothelial cells to maintain the integrity of the monolayer without explicitly considering the mechanical properties of the microenvironment. Furthermore, many models focused on the understanding that the behavior of endothelial monolayer depends on the mechanical properties of the substrate. Using a linear elastic model of the nucleus, cytoplasm, matrix, and VE-cadherin (24), the authors showed that, upon aging, the stiffness of the subendothelial matrix becomes heterogeneous. Recently, models have been developed by combining such mathematical approaches to study angiogenesis, describing the process by which new blood vessels are formed by sprouting from the preexisting vascular bed in healthy and diseased states (25–27). Noting that angiogenesis plays a key role in several physiological and pathological processes, a multiscale model based on the gene expression patterns of individual endothelial cells predicted network growth for varying compositions of the extracellular environment (25). Even though the endothelium expresses abnormal permeability due to the dissociation of adherens junctions during angiogenesis (28), the effect of the dynamics of adherens junctions on vascular homeostasis was not considered in the modeling frameworks.

Thus, in recent times, there has been a renewed interest in developing mathematical models to understand the physiology of the dynamics of adherens junctions. The agent-based model (29) was one of the first models developed to study the effect of mechanical properties of cells to study the dynamics of endothelial permeability. Considering actin stress fibers as viscoelastic springs and adhesion dynamics modeled to follow a catch-slip bond law, the model was able to predict the dynamics of cell-cell junctions. However, the model was limited to 2D cell geometries. A discrete continuum hybrid model (30) was developed to study the effect of calcium waves on cell-cell junctions with 3D endothelial geometries. It was noted that the gaps were higher at the vertices of cells and thus cancer cells extravasate at these locations over edges. The model was able to simulate the opening of gaps but could not simulate the dynamics of the opening and closing of cell-cell junctions. Recently, a continuum model was developed to simulate the three-way feedback between VE-cadherins, RhoA contractility, and Rac-1-derived actin polymerization (31). The myosin-dependent force generation was balanced by the force due to VE-cadherins in addition to being coupled to signaling and cell stress. The role of substrate was not explicitly considered, and hence the model did not study the interplay between the cell-extracellular matrix (ECM) and cell-cell junctions.

The endothelium in vivo experiences several mechanical and chemical stimuli simultaneously. In atherosclerosis, low-density lipoprotein cholesterol, high-density lipoprotein cholesterol, and triglycerides are overexpressed while elevated levels of blood pressure and stiffer arterial matrix are observed to simultaneously affect the behavior of endothelial cells (32). Similarly, the transitioning of endothelial cells to mesenchymal states is found to be affected by the mechanical properties of the ECM in addition to the chemical stimuli such as TGF-β (11). However, the models developed so far have not considered the effect of mechanical stimuli such as traction due to the flow of blood, cell contractility, and hemodynamic pressure, along with chemical stimuli, acting simultaneously on the endothelium. The effect of the interplay between the cell-cell junctions and cell-substrate junctions in regulating endothelial permeability has not been considered either. Thus, the existing mathematical models are unable to predict the behavior of adherens junctions due to changing mechanical and chemical properties of the substrate. In this regard, we have developed a novel continuum-level contact-mechanics-based mathematical model that couples VE-cadherin to Ca^{2+}-based stress fiber contractility and cell-substrate adhesion. VE-cadherins are thus considered mechanical entities that can form and break contact with VE-cadherins from neighboring cells following the principle of a traction-separation law. We use a strain-rate and Ca^{2+} concentration-dependent phenomenological model to compute the contractile stress in an endothelial cell. Next, we define contact between endothelial cells in the monolayer and between the monolayer and the substrate through the traction-separation law. We study the effect of different cell-external mechanical stimuli, such as traction forces due to cell-matrix interactions or shear forces due to blood flow, and randomly varying intercellular normal and shear loads, simulating the cell-internal mechanical stimuli, on endothelial monolayer permeability. Furthermore, for simplicity, we model the substrate as a linear elastic material and use an ad-hoc mechanochemical coupling between the substrate stiffness

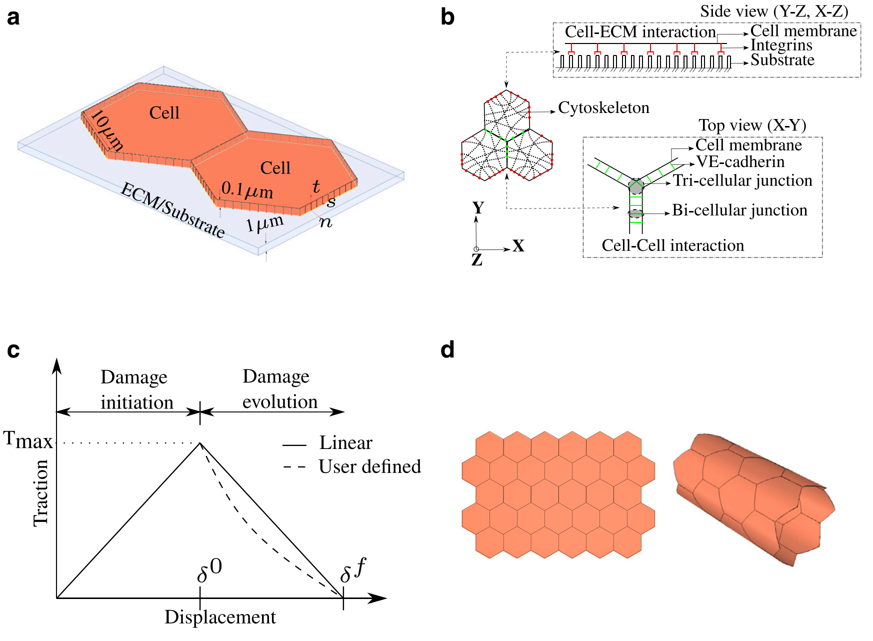

FIGURE 2 (*a*) Schematic of two endothelial cells seeded on an ECM. They form contact with each other and with the ECM. Each wall of the cell coming in contact with the neighboring cell is split into a number of smaller divisions. The ECM is modeled only when we study the effect of the ECM on the endothelial cells. Otherwise, the ECM is neglected. (*b*) Schematic showing the mechanical connection within the cell. A mechanical equilibrium is established between the cytoskeleton, VE-cadherins, and integrins. Cell-cell junctions are formed in the x-y plane while cell-ECM junctions are formed in the x-z and y-z planes. (*c*) Schematic of the traction-separation law. The traction-separation law is modeled as the mechanical equivalent of the catch-slip bond. Damage initiation is assumed to be linear and evolution can be either linear or any other user-defined function. In this article, damage evolution is assumed to be linear. δ^0 indicates the relative displacement at which the damage is initiated in each direction, and δ^f the effective relative displacement at complete damage. (*d*) Schematic of initial geometry of endothelial monolayer considered for analysis. Planar and cylindrical monolayers are considered in the study. The number of cells and curvature of the cylinder can be varied. To see this figure in color, go online.

and the cytoplasmic Ca^{2+} concentration (33). Thus, we study the individual and collective effect of the mechanical and chemical stimuli on endothelial permeability. In addition, we also study the effect of the geometry of the endothelial monolayer on permeability. In the following sections, we introduce the model and show several numerical examples that demonstrate how different mechanical and chemical stimuli affect permeability.

MATERIALS AND METHODS

In this article, we introduce a novel mathematical model to study the dynamic behavior of endothelial cells forming bonds with the neighboring cells in the monolayer and the substrate on which they are seeded, as shown in Fig. 2 *a*. We simplify the system and assume that there exists a mechanical equilibrium between cell-cell interaction (VE-cadherins), cell-substrate interaction (integrins), and the cytoskeleton, as shown in Fig. 2 *b*. Since we model endothelial cells and substrate as 3D geometries, the top view of Fig. 2 *b* (x-y plane) shows cell-cell junctions while the side view (y-z or x-z planes) shows the cell-substrate junctions. In the current continuum modeling framework, the cell-cell and cell-substrate bonds are formulated as contact cohesive surfaces. This allows any two surfaces to lose and form contacts over time, simulate the catch-slip behavior observed in cell-cell junctions (34) and cell-ECM junctions (35), and also analyze 3D geometries, unlike earlier models that focused mainly on 2D geometries. The cell-cell and cell-substrate bonds are modeled to be formed when two surfaces come in contact with each other and broken when they lose contact, determined by the separation distance between them.

The catch-slip bond law that has been used in the literature for simulating cell-cell adhesion dynamics considers a linear combination of catch and slip zones exhibited by the cell-cell adhesion (29). Simulations show that, while stretching the cell-cell junctions, the rate of binding increases up to a particular limit and then reduces. Recent in vitro experiments performed on a single cell-cell junction have shown that the force-displacement curve exhibits a linear increase up to a point and then drops after reaching a peak (36). In this regard, we hypothesize the traction-separation law, as shown in Fig. 2 *c*, as a mechanical equivalent of the catch-slip bond law (explained in detail in VE-cadherin model) and use this on top of cohesive surfaces to dictate how surfaces should damage (disassembly of bonds). Since we do not model individual VE-cadherin-VE-cadherin bonds, we simulate the collective average behavior of many cell-cell junctions over the contact surface through a traction-separation law. Similarly, we also model the cell-substrate interaction following the traction-separation law. The model has been implemented in ABAQUS, which is a commercial finite element solver (37). Files with geometry, material definitions, and other algorithmic implementations (inp, UMAT, UAMP) are distributed as open-source and can be downloaded via the link provided in supporting material, section 1.

Initial geometry and boundary conditions

The geometry of planar and cylindrical endothelial monolayers considered for analysis is shown in Fig. 2 *d*. A portion of the planar monolayer can be seen in Fig. 2 *a*. The initial geometry of the cell is considered to be a regular hexagon with sides of 10 μm and a thickness of 0.1 μm (38). The number of endothelial cells forming the planar and cylindrical monolayers is considered to be a variable. The walls of the cells present on the boundary of the monolayer are fixed (all degrees of freedom constrained). Furthermore, each wall of the cell is split uniformly into a number of smaller divisions, as can be seen in Fig. 2 *a*. The axis normal to the surface is *n*, while the shear axes are indicated by *s* and *t*. The initial condition is assumed to be stress-free, with cells perfectly in contact with the neighboring cells. The distance between the surfaces in contact is taken to be 0 at the stress-free state. A small-sliding contact formulation with cohesive behavior for surface-surface contact is defined, with any secondary node experiencing contact taken to be an eligible secondary node for contact. This allows a given master node to form a cohesive bond with any secondary node that comes in contact and satisfies the traction-separation law, even though those two nodes may not be in contact at the initial state. Normal contact behavior is defined as "hard-contact," preventing the penetration of cells, and allowing separation of surfaces after contact. Whenever the effect of ECM is considered in the analysis (see

permeability increases with ECM stiffness due to mechanochemical coupling and permeability increases with the magnitude of disturbed flow), the ECM is explicitly modeled and cells are assumed to be in contact with the ECM in addition to being in contact with the neighboring cells, as shown in Fig. 2 *a*. The ECM is neglected otherwise. Similar to cell-cell contact, the distance between the cell-ECM surfaces in contact is taken to be 0 at the stress-free state. The parameter values used in the analyses are given in supporting material, section 5.

VE-cadherin model

The traction-separation law for the damage of the cell-cell junction could be split into two zones, one for damage initiation and the other for damage evolution, as can be seen in Fig. 2 *c*, which could be compared with the binding and unbinding zones present in a catch-slip bond law. When the surfaces in contact are stretched away up to δ^0, the bonds stabilize with stretch and the traction force increases. When the stretch reaches δ^0, the damage is said to be initiated and the bond starts to destabilize. Furthermore, stretch of the bond at which there is complete damage is indicated by δ^f. A linear constitutive relation between stress and strain in the VE-cadherin bond before damage initiation is assumed as given in Eq. 1.

$$\begin{Bmatrix} T_n \\ T_s \\ T_t \end{Bmatrix} = \begin{bmatrix} K_{nn} & K_{ns} & K_{nt} \\ K_{sn} & K_{ss} & K_{st} \\ K_{tn} & K_{ts} & K_{tt} \end{bmatrix} \begin{Bmatrix} \varepsilon_n \\ \varepsilon_s \\ \varepsilon_t \end{Bmatrix} \tag{1}$$

where T is the traction, K is the stiffness, and ε is the strain given in the matrix notation. Subscript n indicates the normal direction, and s and t indicate the shear directions. As can be seen from Fig. 2 *c*, damage could be initiated either upon reaching a relative displacement of δ^0 (maximum separation criteria) or a traction of T_{max} (maximum traction criteria). In this article, we use the maximum separation criteria as given in Eq. 2 for the quantitative analyses,

$$max\left\{ \frac{\delta_n}{\delta_n^0}, \frac{\delta_s}{\delta_s^0}, \frac{\delta_t}{\delta_t^0} \right\} = 1 \tag{2}$$

where δ_n^0, δ_s^0, and δ_t^0 are the maximum values of separation allowed for damage initiation in normal and shear directions, and δ_n, δ_s, and δ_t are the current separation values in their respective directions, but can equivalently be analyzed using maximum traction criteria. Once the damage is initiated, its evolution can follow linear, exponential, or any other user-defined response, as can be seen in Fig. 2 *c*. In this article, we use a linear evolution law, where the damage variable D, representing the overall damage in the material (equivalent to slip zone) follows Eq. 3. The initial value of D is 0, which increases to a value of 1 upon complete damage.

$$D = \frac{\delta_m^f \left(\delta_m^{max} - \delta_m^0 \right)}{\delta_m^{max} \left(\delta_m^f - \delta_m^0 \right)} \tag{3}$$

Here, $\delta_m = \sqrt{\delta_n^2 + \delta_s^2 + \delta_t^2}$ is the effective separation. δ_m^f is the effective stretch at complete damage, and δ_m^{max} indicates the maximum value of effective separation attained during the loading history. The effective displacement at the damage initiation (δ_m^0), without loss of generality, assuming $\delta_s^0 = \delta_t^0$, can be evaluated, following (39), as

$$\delta_m^0 = \begin{cases} \delta_n^0 \delta_t^0 \sqrt{\dfrac{1+\beta^2}{\delta_t^{02} + \beta \delta_n^{02}}} & \delta_n > 0 \\ \delta_t^0 & \delta_n \le 0 \end{cases} \tag{4}$$

where $\beta = \frac{\delta_{shear}}{\delta_n}$ and δ_{shear} represents the norm of the vector defining the tangential relative displacements (δ_s and δ_t). Readers are referred to (39) for further details on derivation and analysis. The stress components (T_n, T_s, and T_t) are affected due to damage according to Eq. 5, where ($\overline{T}_n$, $\overline{T}_s$, and $\overline{T}_t$) are the stress components predicted by elastic traction-separation law before damage initiation.

$$\begin{aligned} T_n &= \begin{cases} (1-D)\overline{T}_n & \overline{T}_n \ge 0 \\ \overline{T}_n & otherwise \end{cases} \\ T_s &= (1-D)\overline{T}_s \\ T_t &= (1-D)\overline{T}_t \end{aligned} \tag{5}$$

Thus, using Eqs. 1, 2, 3, 4, and 5, VE-cadherin-based cell-cell adhesion has been modeled in this article, and the behavior is similar to the catch-slip bond observed experimentally. In a catch-slip bond model, by varying the rate of association and dissociation, with force applied on the bond being the independent parameter, a spectrum of bond lifetime versus force curves can be obtained (29). Similarly, by changing the values of separation at damage initiation (δ^0) and at complete failure (δ^f), we can obtain a range of traction-separation curves. Even though there is no direct correlation between the lifetime-force curves obtained using the catch-slip bond law and traction-separation curves obtained via contact mechanics-based traction-separation law, physics simulated by both of them are equivalent. More details of parameter variations of the traction-separation law are given in supporting material, section 2. In this article, the value of bond stiffness is chosen such that the maximum value of contact force is in the same range as the bond force observed experimentally (40). In addition, it is to be noted that the lifetime of a bond is non-zero at an initial state where the applied force is zero, while in the case of traction-separation law the initial condition is chosen to be with a contact force being zero. In addition to cell-cell adhesion, it has been observed that cell-substrate adhesion also behaves as a catch-slip bond (35). Hence, to study the role of cell-substrate contact on permeability in silico, we model the integrin-based cell-substrate bond using traction-separation law.

Cytoskeleton model

The VE-cadherins are further connected to the actin cytoskeleton via the catenin and vinculin family of proteins. To include the effect of the actin cytoskeleton on VE-cadherin bonds, we assume the total stress in the cell to comprise two components, active stress (σ^a) and passive stress (σ^p) (Eq. 6). Active stress is generated by the stress fiber contractility while passive stress is due to other components in the cytoskeleton.

$$\Sigma_{ij} = \sigma_{ij}^p + \sigma_{ij}^a \tag{6}$$

where $i,j = 1,2,3$ are indices, following Einstein summation convention. In this article, for simplicity, the passive stress growth is assumed to follow a linear elastic material law, as given in Eq. 7.

$$\sigma_{ij}^p = C_{ijkl}\varepsilon_{kl} \tag{7}$$

where C_{ijkl} is the stiffness tensor, which depends on Young's modulus E and Poisson's ratio ν. The active part of the cytoskeleton is assumed to be composed of stress fibers oriented in discrete angles, and follows a strain-rate-dependent growth (41), as given in Eq. 8. Readers are referred to (41–43) for more information on the active stress growth model used.

$$\sigma^a(\omega,\varphi) = \eta(\omega,\varphi)\sigma_{max}\left(1 + \frac{K_t\dot{\varepsilon}(\omega,\varphi)}{\sqrt{1+(K_t\,\dot{\varepsilon}(\omega,\varphi))^2}}\right) \tag{8}$$

where $\eta(\omega,\varphi)$ $(0 \le \eta(\omega,\varphi) \le 1)$ is a scalar representing the stress fiber concentration at a particular orientation (ω,φ). The angle that the fiber makes with the x_3 axis is given by ω, and φ is the angle of the projection of fiber on the x_1-x_2 plane with respect to the x_1 axis. K_t is a constant relating the active stress and strain rate, termed strain rate coefficient. Following (41), the growth of stress fiber concentration follows an ordinary differential equation (ODE) (given in Eq. 9)

$$\dot{\eta}(\omega,\varphi) = (1-\eta(\omega,\varphi))\widehat{C}\overline{k}_f - \left(1 - \frac{\sigma^a(\omega,\varphi)}{\eta(\omega,\varphi)\sigma_{max}}\right)\eta(\omega,\varphi)\overline{k}_b \tag{9}$$

where $\overline{k}_f$ and $\overline{k}_b$ are the rates of association and dissociation, respectively and $\widehat{C}$ is the calcium concentration. The maximum stress that the stress fibers can carry is given by σ_{max}. Upon evaluating the active stress in a given stress fiber oriented at a particular orientation (ω,φ), the active Cauchy stress tensor can be evaluated (42,44) as given in Eq. 10

$$\sigma^a_{ij} = \frac{3}{4\pi}\int_0^{2\pi}\int_0^{\pi}\sigma^a(\omega,\varphi)m_i m_j \sin\omega\, d\omega\, d\varphi \tag{10}$$

where, m_i and m_j denote the i and j components of the generalized unit vector in the direction of the given stress fiber. Further details on derivation are given in supporting material, section 3. Upon addition of Eqs. 7 and 10, the total Cauchy stress (Eq. 6) in the cell can be evaluated. The static mechanical equilibrium equation is then solved using a commercial finite element solver ABAQUS. Representative finite element simulations of the numerical examples presented in this article is given in supporting videos S1–S5.

Numerical scheme

The finite element method is used to solve the mechanical equilibrium between the cytoskeletal stress and the contact stress generated at the cell-cell and cell-substrate interactions given by

$$\nabla.\boldsymbol{\Sigma} + \widehat{\boldsymbol{\tau}}_{cc} + \widehat{\boldsymbol{\tau}}_{cs} = 0. \tag{11}$$

Here, $\boldsymbol{\Sigma}$ is the total stress in the cytoskeleton, as given in Eq. 6, $\widehat{\boldsymbol{\tau}}_{cc}$ is the traction force at the cell-cell interface, and $\widehat{\boldsymbol{\tau}}_{cs}$ is the traction force at the cell-substrate interface. Thus, any change to total stress is balanced by the change in cell-cell and cell-substrate traction forces resulting in a connection between the cytoskeleton, VE-cadherins, and integrins, as observed in (45). The geometry is discretized with tetrahedral elements and linear shape functions are used. Numerical integration of Eq. 10 is performed by assuming that the stress fibers are distributed uniformly over the cell domain in 20 discrete angles. Numerical integration of the stress fiber growth ODE is performed via the forward Euler method. In the case of small strain formulation, the consistent Jacobian (**C**) needed for solving the mechanical equilibrium can be evaluated as

$$\mathbf{C} = \frac{\partial\Delta\boldsymbol{\Sigma}}{\partial\Delta\varepsilon} \tag{12}$$

It is to be noted that the Jacobian **C** is not part of the stress update, and is required by the solver only to obtain quadratic convergence of the Newton scheme. Hence, using different methods to evaluate Eq. 12 might only affect the convergence and does not affect the result. As mentioned earlier, in this article, the passive stress growth is assumed to be linear elastic, and small strain finite element analysis is performed. This could easily be replaced by hyperelastic materials such as neo-Hookean and finite strain analysis can be performed. The procedure to be followed to evaluate Jacobian in the case of finite strain formulation is given in supporting material, section 4. The pseudocode of the solution framework is given in supporting material, section 12.

Permeability of the endothelium

The endothelium is said to be permeable to a particular molecule when it is possible for that molecule to cross the endothelial barrier. As explained earlier, in this article, we consider only the VE-cadherin-regulated paracellular pathway to study permeability. This is an important measure of a core function of the endothelium and a key measure of health and disease. Understanding permeability helps in designing effective drug delivery systems. In experiments, permeability is usually measured in two ways: 1) using a dye to measure the amount of tracer molecule that has crossed the endothelium via cell-cell junctions (46) and 2) by measuring the transendothelial electrical resistance (47).

Experiments, in vitro, performed on human aortic endothelial cells (HAECs) estimate the permeability of the monolayer by measuring the amount of FITC-avidin tracer that crosses the monolayer via the paracellular gaps (46). The subendothelial accumulation of the tracer is quantified using a fluorimetric plate reader. The higher amount of tracer collected below the endothelial cell monolayer indicates higher permeability of the monolayer. The tracer molecules are larger than the intracellular pathways, resulting in the quantification of paracellular pathways alone. The size of the tracer molecule used can also indirectly indicate the size of paracellular gaps, which is not possible to be measured using the transendothelial electrical resistance technique.

Thus, for in in silico experiments, we define permeability (χ) as the ratio of the number of open cell-cell junctions (N_o) to the total number of cell-cell junctions (N_t) in the model of a monolayer (Eq. 13). A junction is said to be open if the normal distance between two such surfaces is greater than δ^f_m or, equivalently, if the contact force between two surfaces that were in contact earlier becomes 0. The normal distance between two surfaces can imply the gap size, and therefore variation of permeability with respect to gap size can be studied. Cell-cell junctions in a monolayer can be split into bicellular and tricellular junctions where two and three cells are in contact, respectively, as shown in Fig. 2 *b*. In the simulations presented in this article, as we perform linear static analysis where all deformation measures are evaluated with respect to the initial configuration, bicellular and tricellular junctions are also defined with respect to the initial configuration and are not updated with deformation.

$$\chi = \frac{N_o}{N_t} \tag{13}$$

RESULTS AND DISCUSSION

Permeability increases with contractility

The in vitro experiments performed on planar monolayers use pro-inflammatory agents such as thrombin to induce permeability. The addition of thrombin activates the RhoA/ROCK pathway causing an increase in contractility, which then leads to an increase in permeability (15). In our mathematical model, we simulate such a behavior via the calcium concentration parameter $\widehat{C}$, used to evaluate stress fiber concentration (Eq. 9). Activation of $\widehat{C}$ (non-zero, but uniform over the cell domain) results in the growth of active stress ($\sigma^a(\varphi,\omega)$) and cells undergo uniform contraction. This leads to a uniform strain distribution as shown in Fig. S8 *a*. However, observing the dynamic behavior of endothelial cells in in vitro experiments (29) the location

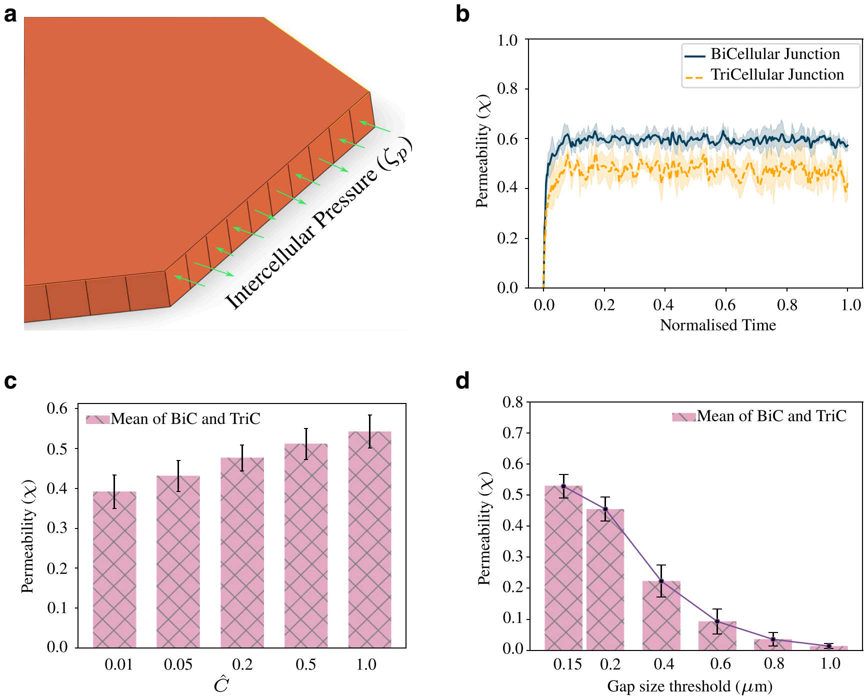

FIGURE 3 Permeability is defined by the ratio of the number of open junctions to the total number of junctions. Error bars indicate mean $\pm$ SD to highlight variability. (*a*) Cell walls are split into 10 divisions and random intercellular pressure is applied to each division. Permeability at a particular location varies based on the local history of loading. (*b*) Variation of permeability over time. Bicellular junctions exhibit higher permeability than tricellular junctions when the only mechanical stimuli that cell-cell junctions experience are the random intercellular pressure loads ($\widehat{C} = 1$, gap size threshold = 0.15 μm). (*c*) Steady-state values of permeability evaluated for changing $\widehat{C}$. As $\widehat{C}$ increases, active stress and intercellular pressure increases, leading to higher permeability (gap size threshold = 0.15 μm). (*d*) With an increase in the gap size threshold, permeability reduces ($\widehat{C} = 1.0$). To see this figure in color, go online.

of the opening of cell-cell junctions varies in a random manner. Random thermal fluctuations and other nonequilibrium cellular processes occur in the cell (48) resulting in a change of shape and mechanical properties. To understand the role of mechanics on such a random behavior, we consider a planar monolayer (Fig. 2 *d*) of hexagonal endothelial cells (Fig. 2 *a*), subject them to different types of mechanical stimuli, and compare permeability. Here, the planar monolayer consists of 32 cells, where each cell is assumed to be in contact with its neighbors at the start of the simulation, and ECM is neglected. The number of cells in the monolayer does not have a significant impact on permeability as long as the number is sufficiently large. We tested this, where 19, 24, 32, or 42 cells did not show much difference in permeability. However, having only 7 cells in the monolayer resulted in large fluctuations in the tricellular permeability, as explained further in supporting material, section 11. This was because of the low number of tricellular junctions available to evaluate the average permeability. Hence, in all the simulations performed on the planar monolayer, we chose 32 cells. Furthermore, to be able to replicate the random opening and closing of cell-cell junctions, we split the walls of each cell into 10 divisions, and apply intercellular pressure loading (ζ_p) of random magnitude on each of these divisions, as shown in Fig. 3 *a*. In our model, the random pressure loading represents the random compressive and tensile stresses generated in the actin filaments leading to self-organized contractility (49), which further causes variation in tension exerted on adherens junctions leading to variation in permeability (15). Dividing the cell wall into smaller divisions and changing the load randomly allowed us to induce random spatial and temporal variations in the cellular stresses. The randomly varying intercellular pressure load (ζ_p) is obtained by multiplying the intercellular pressure load factor ($\widehat{\zeta}_p$) by a random number varying uniformly between -0.5 and 0.5. Furthermore, since ζ_p represents the random tensile and compressive stresses occurring at the cell-cell junctions due to polymerization and contractility, we use an ad hoc nonlinear relation between $\widehat{\zeta}_p$ and $\widehat{C}$, as shown in Fig. S5. The range of values of $\widehat{\zeta}_p$ is chosen such that cell-cell junctions exhibit dynamic behavior and follow the traction-separation law. Based on the history of the loading on the individual node in the finite element mesh, each of them could be either open or closed, resulting in a behavior similar to that observed experimentally. With the application of random pressure loading, we see that the strain distribution is now more heterogeneous with local regions of high and low strain present, as shown in Fig. S8 *b*.

Following the definition given in Eq. 13, we can evaluate the permeability for the planar monolayer undergoing random opening and closing of VE-cadherin bonds. Using a value of $\widehat{C} = 1$ to evaluate the active stress and equivalent intercellular pressure load, we force the cell-cell junctions to open and close randomly following the traction-separation law, which reaches a steady state over time, as seen in Fig. 3 *b*. Investigating the location of permeability, we find that permeability is higher for bicellular junctions compared with tricellular junctions. Since the random pressure loads applied are normal to the surface, the fraction of bicellular junctions that break is

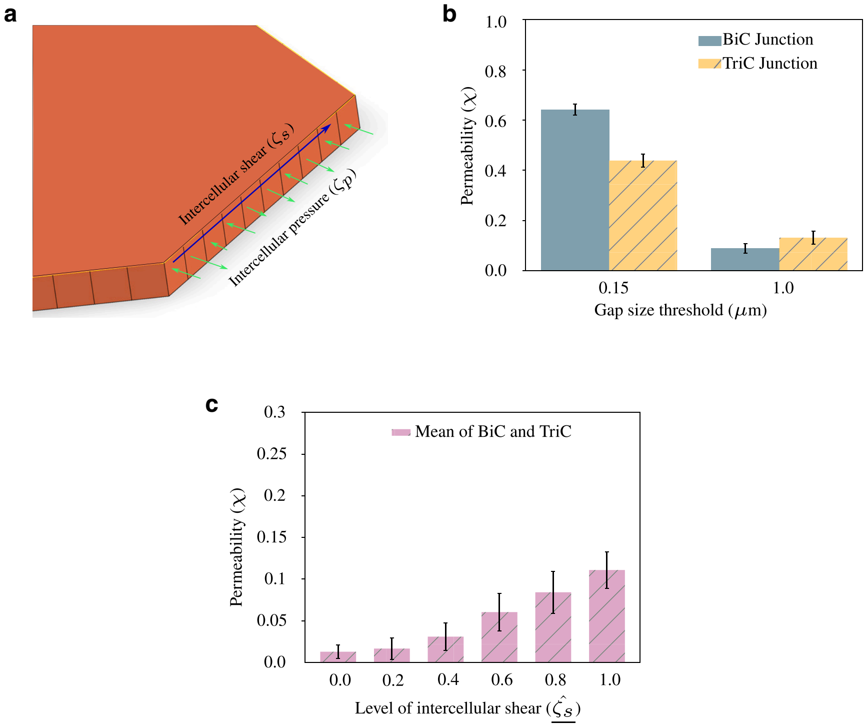

FIGURE 4 (*a*) Schematic of intercellular shear (ζ_s) and pressure (ζ_p) applied on a cell-cell contact surface. Intercellular shear stress is applied as a traction force. (*b*) Due to the application of intercellular shear, the permeability of tricellular junctions is higher than bicellular junctions when the gap size threshold is 1 μm ($\widehat{C} = 1.0$, $\widehat{\zeta}_s = 0.01$ MPa). (*c*) Permeability increases with an increase in the magnitude of intercellular shear. Gap size threshold considered is 1 μm. We only vary intercellular shear, keeping pressure and $\widehat{C}$ constant ($\widehat{C} = 1.0$, $\underline{\widehat{\zeta}_s}$ $(1.0) = 0.01$ MPa). Error bars indicate mean $\pm$ SD to highlight variability. To see this figure in color, go online.

higher than the tricellular junctions resulting in a higher value of χ at bicellular junctions. In vitro experiments performed on endothelial cells showed that Ca^{2+} via RhoA/ROCK activation played a critical role in maintaining barrier integrity (15). To study the effect of contractility on permeability, we vary the value of $\widehat{C}$, and find that permeability also changes accordingly. Our simulations showed that permeability is around 0.4 for $\widehat{C} = 0.01$ and increases to 0.55 for $\widehat{C} = 1$, matching the experimental observations qualitatively (15). The values of permeability presented in Fig. 3 *c* are evaluated by calculating the average of the last 100 data points (iterations) of the mean of bicellular and tricellular junction permeability. Thus, our simulations indicate an intricate interplay between the Ca^{2+} signaling, intercellular stresses acting on adherens junctions, and permeability, which needs to be studied in detail experimentally.

Furthermore, we study the variation of permeability by changing the threshold of the normal distance between cells that defines when a gap is considered open. We hypothesize that this is equivalent to changing the size of tracer molecules in an in vitro experiment. Our simulations show that, as the criterion for a gap to be considered open is increased, permeability reduces. We found that this drop is nonlinear, as can be seen in Fig. 3 *d*. This shows that the permeability of an endothelial monolayer to a 1 μm particle is almost 10 times lower than that of a 0.15 μm particle and 4 times that of a 0.4 μm particle. Thus, with this simulation, based on the size of the leukocyte (in vivo) or tracer molecule (in vitro), we can estimate the chance of crossing the endothelial barrier via the paracellular pathway.

Location of higher permeability varies with mechanical stimuli

Normal and shear stresses between the cells dictate the direction of motion of individual cells in a monolayer, termed plithotaxis (22,50). It was observed that leader cells induced normal strains on the follower cells in the rear and shear strains to the cells by the side. This resulted in coordinated traction forces leading to coordinated cell migration (50). In addition, in vitro experimental studies have also shown that the difference in permeability between bi- and tricellular junctions depends on the type of cell, passage number, and tracer molecule used. It was observed that, while studying permeability with HAECs, bicellular junctions showed less involvement compared with that with porcine aortic endothelial cells, and found that during experiments with HAECs a majority of tracer molecules accumulated below tricellular junctions (46). We, therefore, hypothesize that the permeability of the endothelial monolayer depends on the mechanical forces that individual cells in a monolayer experience. Hence, to study the effect of mechanical forces on endothelial permeability, we apply intercellular shear stress as traction force at cell-cell junctions with randomly varying magnitudes in addition to random intercellular pressure loading, as shown in Fig. 4 *a*.

We evaluate intercellular shear stress (ζ_s) as a product of intercellular shear load factor ($\widehat{\zeta}_s$) and a random number

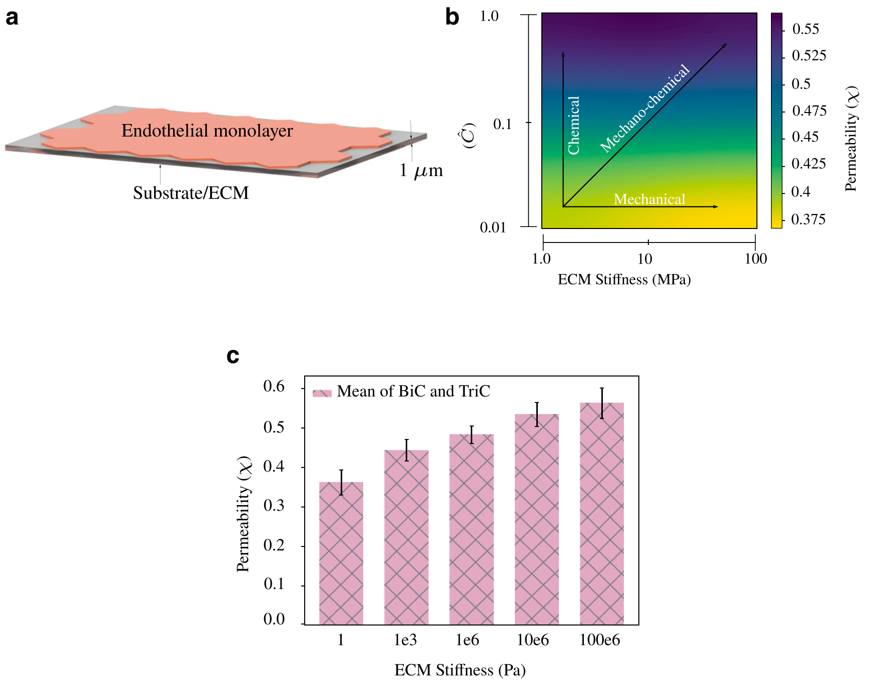

FIGURE 5 (*a*) Schematic of an endothelial monolayer consisting of 32 cells forming a contact with the ECM below. The traction-separation law is applied to cell-cell and cell-ECM contacts. (*b*) Heatmap of permeability for purely mechanical (*rows*), purely chemical (*columns*), and mechano-chemical (*diagonal*) effect. An increase in ECM stiffness along with an increase in calcium concentration, which induces higher contractility, is necessary to simulate the effect of ECM stiffness on permeability observed experimentally. (*c*) With an increase in ECM stiffness along with mechanochemical coupling (*diagonal elements*), the monolayer exhibits higher permeability. $\widehat{C}$ = [0.01, 0.05, 0.2, 0.5, 1.0] for ECM = [1, 1e-3, 1e-6, 10e-6, 100e-6] Pa, respectively. Error bars indicate mean ± SD to highlight variability. To see this figure in color, go online.

varying uniformly between −0.5 and 0.5. The magnitude of $\widehat{\zeta}_s$ is chosen such that cells are able to exhibit dynamic opening and closing of cell-cell junctions following the traction-separation law. Upon applying intercellular shear stress of random magnitudes at each iteration, and keeping the gap size threshold to be 1 μm, we find that permeability is higher at tricellular junctions compared with bicellular junctions. But when the gap size threshold is reduced to 0.15 μm, the permeability is higher at bicellular junctions, as seen in Fig. 4 *b*, similar to the condition without shear stress discussed in permeability increases with contractility. It was further observed that, if the shear between the cells was absent, the permeability of bicellular junctions was higher than that of tricellular junctions even when the gap size threshold was 1 μm. Simulations showed that the shear stress between cells leads to twisting type behavior of endothelial cells, resulting in the observed differences between bicellular and tricellular junctions. We also study the effect of the varying magnitude of shear between cells and find that, as the magnitude increases, permeability also increases for a given gap size threshold, as seen in Fig. 4 *c*. These observations imply that the chance of transcellular migration occurring at tricellular junctions or bicellular junctions depends on the mechanical stimuli. The simulations thus provide an experimentally testable hypothesis that, when the physiology and functioning of the cells are such that cells experience intercellular shear stress, larger particles transmigrate at tricellular junctions while they prefer bicellular junctions in the absence of intercellular shear stress. The understanding can be further translated to engineer the location of permeability by manipulating the mechanical stimuli that the endothelium experiences. This would help in optimizing drug delivery techniques for the many drugs that show promising results in vitro, but that fail to cross the endothelial barrier and therefore do not reach the desired location of diseases.

Permeability increases with ECM stiffness due to mechanochemical coupling

Recent experimental studies indicate that cardiovascular and many other diseases are associated with variations in the mechanical properties of the ECM. Biomechanical tests have found that atherosclerotic plaques are stiffer than endothelial basement membranes by several orders of magnitude (51,52). The mechanical properties of the ECM are also found to be responsible for endothelial-mesenchymal transition, which is a characteristic of cancer extravasation occurring via the endothelium exhibiting abnormal permeability (11). To study permeability, experiments in vitro involve culturing cells on a substrate and using pro-inflammatory agents to induce permeability. Several in vitro experiments have shown the relation between substrate properties, both mechanical (18,53) and geometrical (19,54), on permeability. Cells, when attached to the ECM, form focal adhesions consisting of integrins, which transfer forces to and from the cell. Downstream, focal adhesions are connected to the cytoskeleton machinery, which is further connected to VE-cadherins. Hence, VE-cadherin behavior is regulated by integrin behavior and vice versa. Accordingly, it has been observed that, with an increase in ECM stiffness, the contractility of the cells increases (55), as does the permeability of the monolayer (18).

In this regard, we consider an ECM below the planar monolayer, as shown in Fig. 5 *a*. For simplicity, in this

article, the ECM is considered to be linear elastic. We model the cell-ECM interaction using the traction-separation law, similar to that of cell-cell interaction, with the parameters used for cell-ECM contact assumed to be the same as those of cell-cell contact. To begin with, we change the ECM stiffness and keep all other parameters fixed to find that permeability reduces with an increase in stiffness, as shown in Fig. 5 *b*. This reduction is due to the higher equivalent stiffness of the cell-ECM bond with higher ECM stiffness. However, experimental observations showed that contractility (55) and permeability increase with the stiffness of ECM (18). Hence, a multiparameter analysis was carried out by changing $\widehat{C}$ and ECM stiffness simultaneously. It could be seen in the heatmap Fig. 5 *b* that neither a purely mechanical effect (row-wise comparison) nor a purely chemical effect (column-wise comparison) simulate the right behavior of cells. It is to be noted that, even though a purely chemical effect exhibits increasing permeability, it would be independent of ECM stiffness, which we consider here as a mechanical effect. Hence a mechanochemical coupled model is necessary to simulate the behavior of cells to varying ECM stiffness (diagonal elements in the heatmap). In this article, we do not model a detailed signaling network connecting the mechanosensors at the cell-ECM junction and the cytoskeleton; however, we choose an ad hoc nonlinear relation between $\widehat{C}$ and ECM stiffness, as shown in Fig. S6. Thus, we find that, with an increase in ECM stiffness, cells respond by increasing active stress and intercellular pressure due to higher contractility, leading to elevated levels of permeability, as shown in Fig. 5 *c*. The simulations thus highlight the importance of mechanochemical signaling pathways leading to a cross talk between the cell-cell and cell-ECM junctions through the cytoskeletal network.

Permeability increases with the magnitude of disturbed flow

Blood vessels in vivo experience several stimuli simultaneously; shear stress, hemodynamic pressure, the tension on the walls, and other local stresses due to geometric effects. In a healthy vascular network, the mechanochemical equilibrium of endothelial cells, under the influence of such forces, is necessary for its proper functioning. Any changes to the equilibrium lead to physiological growth and remodeling to maintain homeostasis (56). The inability to remodel the tissues results in an undesired stress state and makes individuals susceptible to cardiovascular diseases (57). Atherosclerosis is one of the very common cardiovascular diseases and is responsible for the death of millions of people across the world. Abnormal permeability in the endothelium results in the deposition of fat molecules and cholesterol in between the endothelium and surrounding smooth muscle cells (32). This results in the formation of atherosclerotic plaques that are very stiff and reduce the diameter of the vessel causing obstruction to the flow of blood in the blood vessel. It has been observed in several in vivo and in vitro experiments that atherosclerotic plaques are mainly found in the region where blood flow is disturbed, which is common in branching points of the vessel (58,59). To this end, to study the impact of disturbed flow on permeability, we consider a planar monolayer consisting of 32 endothelial cells seeded on a flat substrate and apply randomly varying traction force on top of the monolayer, simulating shear stress due to disturbed blood flow in the endothelium, as shown in Fig. 6 *a*. In in vitro or in vivo models, the distribution of shear stress due to disturbed flow follows complex patterns (60) and the magnitude usually varies on the length scale of several endothelial cells (61). However, for simplicity, we assume that the traction force is uniaxial and changes its magnitude on the length scale of a single cell. When applying our model to specific experiments, the exact force distribution due to disturbed flow patterns can be obtained either using fluid-structure-interaction simulations (62) or through experiments with controlled disturbed flows (63). Thus, we evaluate the random traction force due to flow (ζ_f) as a product of the traction force load factor ($\widehat{\zeta}_f$) and a random number varying uniformly between -0.5 and 0.5. The range of $\widehat{\zeta}_f$ is chosen such that the deformations are within the limit for cell-cell and cell-ECM contacts to follow the traction-separation law. With this setup, we study the effect of variation of the magnitude of traction force acting on the monolayer and the ECM stiffness on permeability by varying the magnitude of $\widehat{\zeta}_f$ from 0.1 to 10 kPa and ECM stiffness from 1 Pa to 1 MPa. The value of $\widehat{C}$ varies with ECM stiffness, as described in Fig. 5 *c*. Furthermore, for simplicity, we assume that there is no intercellular shear stress in the monolayer ($\zeta_s = 0$). We found that permeability increased by the application of random traction forces on the endothelial monolayer. With an increase in the magnitude of the traction force, permeability increased further, as seen in Fig. 6 *b*. In addition, when we increased the ECM stiffness along with the random traction force, the effect on permeability was higher. Results suggest that, as the magnitude of the traction force on the endothelium increases, permeability increases, leading to an increased plaque formation. The higher stiffness of plaques further increases permeability, thereby causing a positive feedback loop. Even though the permeability of the endothelial monolayer increases with the magnitude of traction force and ECM stiffness, we found that, as the ECM stiffness increases, the difference in permeability between endothelial monolayers experiencing traction ($\widehat{\zeta}_f = 10$ kPa) and without traction ($\widehat{\zeta}_f = 0$) reduces, as shown in Fig. 6 *c*. Thus, the simulations suggest that, when the ECM stiffness is low, permeability is dominated by the magnitude of the traction that the endothelium experiences, and, as the ECM stiffness increases, permeability is dominated by the effect of ECM stiffness. We can also observe that, for constant traction force, permeability increases with an increase in ECM

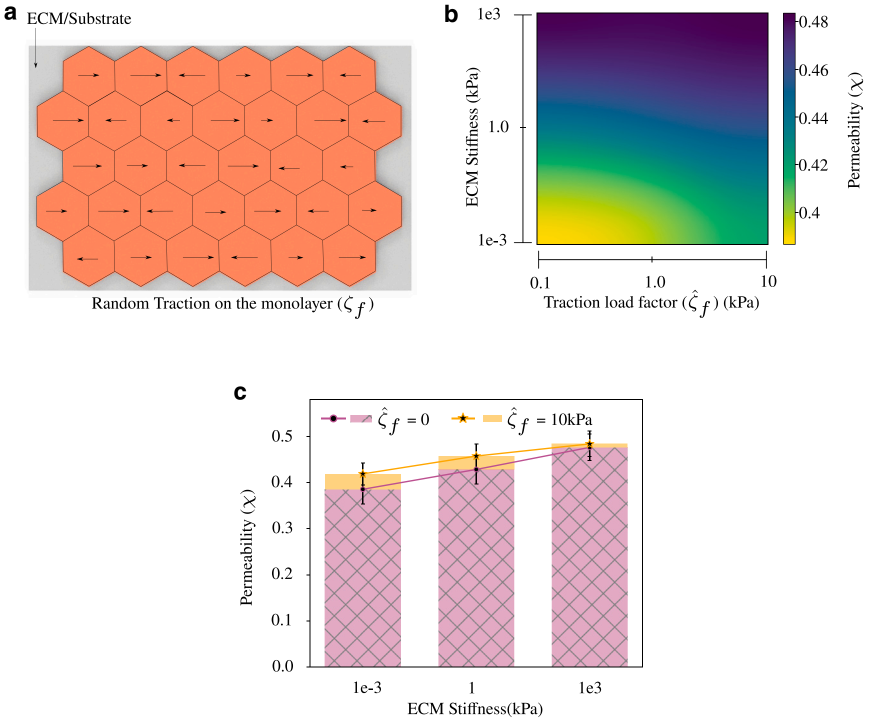

FIGURE 6 (*a*) Schematic of an endothelial monolayer on an ECM subjected to random uniaxial traction force. The magnitude of traction force is randomly varying over time and is different on each cell. $\widehat{C}$ varies with ECM stiffness following Fig. 5 c. (*b*) Heatmap of variation of permeability for changing $\widehat{\zeta}_f$ and ECM stiffness indicates that permeability increases with traction acting on the monolayer and ECM stiffness. (*c*) As the ECM stiffness increases, the difference in permeability between endothelial monolayers with $\widehat{\zeta}_f = 10$ kPa and $\widehat{\zeta}_f = 0$ reduces. Error bars indicate mean $\pm$ SD to highlight variability. To see this figure in color, go online.

stiffness, as shown in Fig. 6 *b*. This indicates that, as humans age and the ECM becomes stiffer (64), the susceptibility to diseases such as atherosclerosis increases. In addition, we compared the effect of random traction, as presented here, and uniform traction, where all cells experience the same traction force, on permeability. Simulations showed that the application of uniform traction force reduced the permeability of the monolayer compared with that of the monolayer experiencing random traction forces. This is presented in supporting material, section 9.

The cylindrical monolayer exhibits higher permeability than the planar monolayer

Usually in vitro experiments are performed by seeding endothelial cells on a petri dish or a glass slide where they form a planar monolayer. But, in vivo, endothelial cells form cylindrical-shaped vascular tubes that allow for the transport of blood. In addition, they are surrounded by the ECM, which alters the properties and behavior of the endothelium. Hence, to extrapolate experimental observations from a planar monolayer to a vascular tube, that is, to be able to predict in vivo behavior based on in vitro experiments, we need to understand the difference in the behavior of cells due to changing geometrical conditions. Using the model presented in this article, we study the role of the geometry of the endothelial monolayer on permeability.

To begin with, we form a cylindrical monolayer with 3D endothelial cells without any ECM surrounding it, as shown in Fig. 7 *a*. The ends of the vessel are fixed to prevent rigid body motion. We study permeability in the cylindrical monolayers due to calcium-based contractility and its associated active stress and intercellular pressure growth, and by neglecting intercellular shear stress and traction force due to disturbed flow ($\widehat{C} = 1.0$, $\zeta_s = 0$, $\zeta_f = 0$). To study the effect of curvature ($\tilde{\kappa}$) of a cylindrical monolayer on permeability, we consider three monolayers with curvatures (evaluated as 1/radius), $\tilde{\kappa} = 0.06\ 1/\mu$m, $0.04\ 1/\mu$m, $0.03\ 1/\mu$m, and compare them with a planar monolayer with $\tilde{\kappa} = 0$. Simulations showed that cylindrical monolayers exhibited higher permeability than planar monolayers for the same set of parameters, as can be seen in Fig. 7 *b*. We also found that, as the curvature of the cylindrical monolayer increased, permeability too increased, in line with the theoretical observations made in the literature (30). Compared with planar monolayers, cylindrical monolayers took longer to reach the steady state, and the value reached was higher also. We observed that higher permeability arises mainly due to the additional degree of freedom that cells enjoy due to curved geometry. By restraining this degree of freedom, which is equivalent to a rigid ECM surrounding the cylindrical monolayer to which the cells are firmly attached, we saw that the permeability of the cylindrical monolayer reduced, and reached a steady state earlier than the case without radial constraint, as seen in Fig. 7 *c*. These simulations show that permeability observed in in vitro experiments performed on a planar monolayer of endothelial cells is not directly applicable to cylindrical monolayers. The results from the simulations yield relationships between the permeabilities of cylindrical monolayers with different curvatures.

This can have implications for understanding the correlation between permeability and the geometry of the blood vessels in healthy and diseased states. The simulations can also help in designing experiments of cylindrical monolayers based on the data available on planar monolayers.

CONCLUSION AND FUTURE WORK

In this article, we have introduced a novel continuum-level modeling framework for studying the dynamic behavior of adherens junctions regulated by VE-cadherins. The traction-separation law as a phenomenologically equivalent mechanism for catch-slip bond law has been used to model the opening and closing of cell-cell junctions. The stiffness of the VE-cadherin bond and the stretch upon which the VE-cadherin bond dissociation is initiated and completely dissociated are the parameters needed to define the law, which are directly available in the literature (e.g., (40)). The cytoskeleton of endothelial cells is assumed to be made up of linear elastic passive component and nonlinear strain-rate-dependent active components. The ODE for stress fiber concentration growth determines the level of active stress in the cytoskeleton. Through the mechanical equilibrium between the stress in the cytoskeleton and the force applied by cell-cell contact and cell-ECM, mechanical feedback between them can be established.

We define permeability as the ratio of the number of open junctions to the total number of adherens junctions present in the monolayer. The model has been able to predict the variation in permeability due to varying gap sizes and multiple mechanical stimuli acting simultaneously. Numerical simulations have been performed to study how using dyes of different sizes shows varying levels of permeability. The size of the gap present in the monolayer might be smaller than the molecular size of the dye resulting in reduced extravasation. Thus, the model can be used to correlate the quantification of VE-cadherin staining and permeability. Simulations showed that the intercellular shear stress played a prominent role in regulating VE-cadherin bonds and hence the permeability of the monolayer. Since mechanical stimuli and mechanical properties vary with endothelial cell types, the simulations give an insight into differences observed in in vitro experiments performed with different cell types and passage numbers. Thus, through the in silico experiments, we are able to develop testable hypotheses on the mechanochemical conditions that result in higher permeability. Simulations also showed that permeability increases with calcium concentration-induced contractility. The role of substrate stiffness on permeability was studied by attaching cells to a linear elastic substrate. It was seen that an increase in substrate stiffness results in lowering permeability. However, adding an ad hoc mechanochemical coupling showed that permeability increases with substrate stiffness. Even though the mechanochemical coupling used in this article is a very simple one, this provides a perfect platform to further study the role of varying mechanical and geometric properties of the substrate on permeability. The linear elastic substrate could be further replaced by collagen-type fibrous material and a detailed mechanochemical coupling pathway could be added easily. In this article, for simplicity, we assumed the parameters of the traction-separation law for cell-ECM contact to be the same as that of cell-cell contact. In future applications of our model, a deeper analysis of cell-ECM contact and the distinction between cell-ECM and cell-cell contacts will be carried out that takes into account the specific properties of the ECM and the endothelium used in specific experiments to be modeled.

It was interesting to note that the geometry of blood vessels determined the probability of the location of atherosclerotic plaques. They were commonly found near the branches of valves where the blood flow is in a disturbed state (65). In numerical simulations, we could study the effect of such a disturbed flow on permeability and its dependence on ECM stiffness by applying random traction forces on endothelial cells in contact with a substrate. Results showed that applying random traction forces increases permeability, and this effect increases with ECM stiffness. Numerical studies also showed that susceptibility to cardiovascular diseases increases with aging due to increased ECM stiffness and the associated increase in permeability of the endothelium. Studies also showed that the cylindrical monolayer, which is similar to endothelium in vivo, exhibits higher permeability than the planar monolayer when there is no ECM surrounding it and behaves like a planar monolayer when surrounded by a rigid ECM. In addition, we also found that permeability increases with the curvature of the cylindrical monolayer and we show through these simulations that the experiments performed in vitro on a planar monolayer do not directly correlate to the behavior in vivo, and the geometry of the endothelium plays a prominent role in determining permeability. Thus, through the numerical simulations presented in this article, we have been able to develop a few experimentally testable hypotheses:

1) The permeability of the cylindrical endothelial monolayer increases with curvature.
2) Intercellular shear stress leads to increased levels of permeability and causes larger gaps at tricellular junctions.
3) Permeability increases with aging due to higher ECM stiffness and the effect of higher ECM stiffness dominates the effect of disturbed flow on permeability.

The model could be thus used to understand the role of chemical and mechanical cues and test the conditions resulting in extravasation of leukocytes in vivo or a dye in vitro, and thereby aid in the design of experiments. The modeling framework can also be used to test under what conditions drug molecules can extravasate and through either tricellular

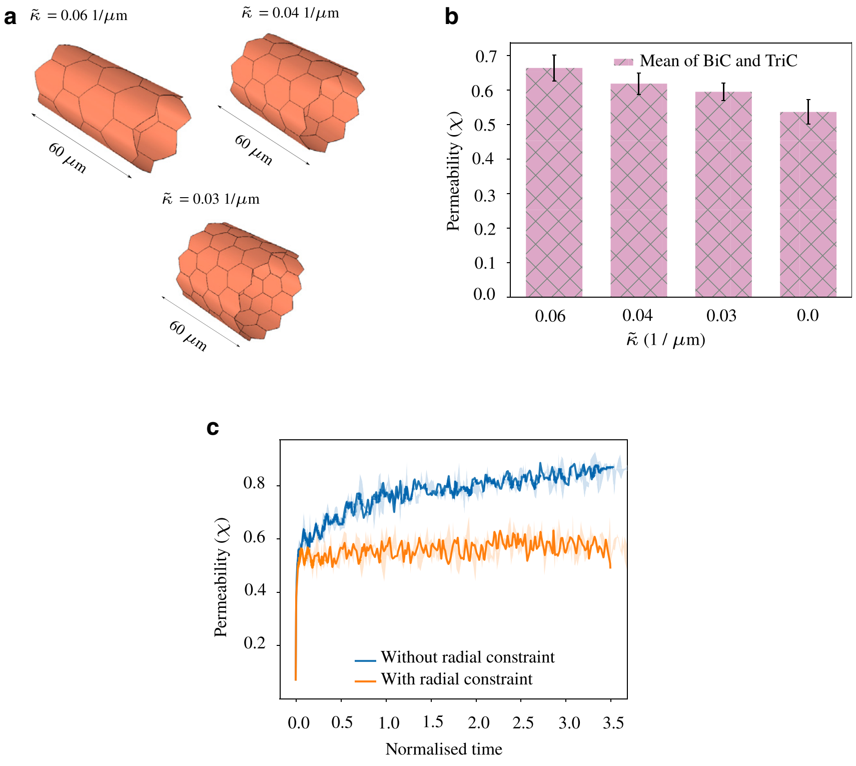

FIGURE 7 (*a*) Cylindrical monolayers with different curvatures (evaluated as 1/radius) ($\tilde{\kappa}$). All cylindrical monolayers have the same length but different radii. Hence, they have different numbers of cells (24,36,48) ($\widehat{C} = 1.0, \zeta_s = 0, \zeta_f = 0$). (*b*) As the curvature reduces, the permeability reduces as well. The planar monolayer has the least permeability compared with cylindrical monolayers. The mean value is obtained by averaging the last 100 data points. Error bars indicate mean ± SD to highlight variability. (*c*) The permeability of a cylindrical monolayer is higher than that of a planar monolayer due to the availability of additional degrees of freedom for its deformation. Upon constraining the radial deformation of cells, permeability is lower and is similar to that of a planar monolayer ($\tilde{\kappa}$ = 0.06 1/μm). To see this figure in color, go online.

or bicellular junctions. This can have several important applications in drug delivery. The model could also be used to determine the right mechanical properties of the ECM that can regulate permeability. This could be used as a method to stop the positive feedback between the ECM and cell contractility resulting in reduced permeability. While the current treatment techniques involve changing the properties of blood using drugs such as statins or using stents to regulate the diameter of arteries (32), the model could be particularly used in finding the right mechanical and chemical environment necessary for either maintaining dynamic homeostasis or rectifying the properties of the ECM such that the cells go back to a healthy state. In addition to atherosclerosis, this model could be used to bolster the antifibrotic treatment techniques used in several diseases such as cancer and liver diseases (66), which helps in regulating the permeability of the endothelium and thereby maintain vascular homeostasis. Furthermore, the model could be used to effectively engineer the vascular network in vitro by studying the role of a hydrogel on vascular permeability (67). The model could also in future be extended to understand the role of aging on vascular stiffening (68) or the effect of cytoskeletal metabolic memory (69) on cell-cell junctions and thereby permeability. The phenomenological model presented in this article is motivated by the mechanical responses observed during in vitro experiments. The formulation of the model is such that it is possible to extend it to include detailed mechanochemical coupling pathways that affect permeability. The traction-separation law which has displacement as the only factor determining when the bonds dissociate completely can include the concentration of VE-cadherin or other proteins as the additional factor that determines the dissociation of VE-cadherin bonds. Simulations showed that permeability was slightly higher in the regions experiencing disturbed flow-type traction forces compared with regions where traction forces are uniform, as shown in supporting material, section 9. We believe that the low increase in permeability with disturbed flow-type conditions is due to the absence of mechanosensors such as glycocalyx that further regulate the cytoskeleton downstream and vary permeability (70,71). Thus, the model presented in this article provides a perfect platform for several extensions and mechanochemical studies.

SUPPORTING MATERIAL

Supporting material can be found online at https://doi.org/10.1016/j.bpj.2023.12.026.

AUTHOR CONTRIBUTIONS

Conceptualization, P.K. and F.S.; code development, P.K.; analysis, P.K.; visualization, P.K.; writing – original draft, P.K.; writing – review & editing, F.S.; supervision, F.S.

ACKNOWLEDGMENTS

We would like to acknowledge the funding from BBSRC grant BB/V002708/1 and UKRI Future Leaders Fellowship (MR/T043571/1). We

would also like to thank Giovanni Guglielmi for discussions about using friendly colors in plots. We also thank the supercomputing facility at the University of Birmingham, BEAR, for constant support with computational requirements.

DECLARATION OF INTERESTS

The authors declare no competing interest.

REFERENCES

1. Lüscher, T. F., and M. Barton. 1997. Biology of the Endothelium. *Clin. Cardiol.* 20:3–10.
2. Yuan, S. Y., and R. R. Rigor. 2010. Regulation of Endothelial Barrier Function. Morgan & Claypool Life Sciences, San Rafael (CA).
3. SEER Training Modules.. Classification & Structure of Blood Vessels. https://training.seer.cancer.gov/anatomy/cardiovascular/blood/classification.html.
4. Tuma, P. L., and A. L. Hubbard. 2003. Transcytosis: Crossing cellular barriers. *Physiol. Rev.* 83:871–932.
5. Komarova, Y., and A. B. Malik. 2010. Regulation of endothelial permeability via paracellular and transcellular transport pathways. *Annu. Rev. Physiol.* 72:463–493.
6. Claesson-Welsh, L., E. Dejana, and D. M. McDonald. 2021. Permeability of the Endothelial Barrier: Identifying and Reconciling Controversies. *Trends Mol. Med.* 27:314–331.
7. Azzi, S., J. K. Hebda, and J. Gavard. 2013. Vascular permeability and drug delivery in cancers. *Front. Oncol.* 3:211, AUG:1–14.
8. Dell'Omo, G., G. Penno, …, R. Pedrinelli. 2004. Abnormal capillary permeability and endothelial dysfunction in hypertension with comorbid Metabolic Syndrome. *Atherosclerosis.* 172:383–389.
9. Khor, Y. H., A. K. Y. Teoh, …, E. H. Walters. 2009. Increased vascular permeability precedes cellular inflammation as asthma control deteriorates. *Clin. Exp. Allergy.* 39:1659–1667.
10. Xu, S. W., I. Ilyas, and J. P. Weng. 2023. Endothelial dysfunction in COVID-19: an overview of evidence, biomarkers, mechanisms and potential therapies. *Acta Pharmacol. Sin.* 44:695–709, –15.
11. Zamani, M., Y. H. Cheng, …, N. F. Huang. 2022. Single-Cell Transcriptomic Census of Endothelial Changes Induced by Matrix Stiffness and the Association with Atherosclerosis. *Adv. Funct. Mater.* 32, 2203069.
12. Birdsey, G. M., N. H. Dryden, …, A. M. Randi. 2008. Transcription factor erg regulates angiogenesis and endothelial apoptosis through VE-cadherin. *Blood.* 111:3498–3506.
13. Gory-Fauré, S., M. H. Prandini, …, P. Huber. 1999. Role of vascular endothelial-cadherin in vascular morphogenesis. *Development.* 126:2093–2102.
14. Dorland, Y. L., and S. Huveneers. 2017. Cell–cell junctional mechanotransduction in endothelial remodeling. *Cell. Mol. Life Sci.* 74:279–292.
15. Kugelmann, D., L. T. Rotkopf, …, J. Waschke. 2018. Histamine causes endothelial barrier disruption via Ca2+-mediated RhoA activation and tension at adherens junctions. *Sci. Rep.* 8:13229.
16. Rabiet, M. J., J. L. Plantier, …, E. Dejana. 1996. Thrombin-induced increase in endothelial permeability is associated with changes in cell-to-cell junction organization. *Arterioscler. Thromb. Vasc. Biol.* 16:488–496.
17. Wang, L., and S. M. Dudek. 2009. Regulation of vascular permeability by sphingosine 1-phosphate. *Microvasc. Res.* 77:39–45.
18. Andresen Eguiluz, R. C., K. B. Kaylan, …, D. E. Leckband. 2017. Substrate stiffness and VE-cadherin mechano-transduction coordinate to regulate endothelial monolayer integrity. *Biomaterials.* 140:45–57.
19. Nakayama, K. H., V. N. Surya, …, N. F. Huang. 2016. Nanoscale Patterning of Extracellular Matrix Alters Endothelial Function under Shear Stress. *Nano Lett.* 16:410–419.
20. Tian, Y., G. Gawlak, …, K. G. Birukov. 2016. Activation of Vascular Endothelial Growth Factor (VEGF) receptor 2 mediates endothelial permeability caused by cyclic stretch. *J. Biol. Chem.* 291:10032–10045.
21. Tikhomirova, A. E., and V. A. Volpert. 2007. Nonlinear dynamics of endothelial cells. *Appl. Math. Lett.* 20:163–169.
22. Szabó, A., R. Ünnep, …, A. Czirók. 2010. Collective cell motion in endothelial monolayers. *Phys. Biol.* 7:046007.
23. Vitorino, P., M. Hammer, …, T. Meyer. 2011. A Steering Model of Endothelial Sheet Migration Recapitulates Monolayer Integrity and Directed Collective Migration. *Mol. Cell Biol.* 31:342–350.
24. Kohn, J. C., T. Abdalrahman, …, T. Franz. 2018. Endothelial cells on an aged subendothelial matrix display heterogeneous strain profiles in silico. *Biomech. Model. Mechanobiol.* 17:1405–1414.
25. Stepanova, D., H. M. Byrne, …, T. Alarcón. 2021. A multiscale model of complex endothelial cell dynamics in early angiogenesis. *PLoS Comput. Biol.* 17:10080555–e1008144.
26. Pillay, S., H. M. Byrne, and P. K. Maini. 2017. Modeling angiogenesis: A discrete to continuum description. *Phys. Rev. E.* 95:012410.
27. McDougall, S. R., A. R. A. Anderson, …, J. A. Sherratt. 2002. Mathematical modelling of flow through vascular networks: Implications for tumour-induced angiogenesis and chemotherapy strategies. *Bull. Math. Biol.* 64:673–702.
28. Nagy, J. A., L. Benjamin, …, H. F. Dvorak. 2008. Vascular permeability, vascular hyperpermeability and angiogenesis. *Angiogenesis.* 11:109–119.
29. Escribano, J., M. B. Chen, …, F. Spill. 2019. Balance of mechanical forces drives endothelial gap formation and may facilitate cancer and immune-cell extravasation. *PLoS Comput. Biol.* 15:10063955–e1006421.
30. Nieto, A., J. Escribano, …, M. J. Gomez-Benito. 2020. Finite element simulation of the structural integrity of endothelial cell monolayers: A step for tumor cell extravasation. *Eng. Fract. Mech.* 224, 106718.
31. McEvoy, E., T. Sneh, …, V. B. Shenoy. 2022. Feedback between mechanosensitive signaling and active forces governs endothelial junction integrity. *Nat. Commun.* 13:7089–7114.
32. Bergheanu, S. C., M. C. Bodde, and J. W. Jukema. 2017. Pathophysiology and treatment of atherosclerosis: Current view and future perspective on lipoprotein modification treatment. *Neth. Heart J.* 25:231–242.
33. Kim, T. J., J. Seong, …, Y. Wang. 2009. Substrate rigidity regulates Ca2+ oscillation via RhoA pathway in stem cells. *J. Cell. Physiol.* 218:285–293.
34. Rakshit, S., Y. Zhang, …, S. Sivasankar. 2012. Ideal, catch, and slip bonds in cadherin adhesion. *Proc. Natl. Acad. Sci. USA.* 109:18815–18820.
35. Novikova, E. A., and C. Storm. 2021. Evolving roles and dynamics for catch and slip bonds during adhesion cluster maturation. *Phys. Rev. E.* 103:032402.
36. Esfahani, A. M., J. Rosenbohm, …, R. Yang. 2021. Characterization of the strain-rate–dependent mechanical response of single cell–cell junctions. *Proc. Natl. Acad. Sci. USA.* 118, e2019347118.
37. Smith, M. 2009. ABAQUS/Standard User's Manual, Version 6.9. Dassault Systèmes Simulia Corp, United States.
38. Félétou, M. 2011. The Endothelium: Part 1: Multiple Functions of the Endothelial Cells—Focus on Endothelium-Derived Vasoactive Mediators. Morgan & Claypool Life Sciences, San Rafael (CA).
39. Camanho, P. P., and C. G. Davila. 2002. Mixed-Mode Decohesion Finite Elements for the Simulation of Delamination in Composite Materials. Technical Report June, NASA. http://citeseerx.ist.psu.edu/viewdoc/download?doi=10.1.1.8.267&rep=rep1&type=pdf.

40. Panorchan, P., J. P. George, and D. Wirtz. 2006. Probing Intercellular Interactions between Vascular Endothelial Cadherin Pairs at Single-molecule Resolution and in Living Cells. *J. Mol. Biol.* 358:665–674.
41. Keshavanarayana, P., M. Ruess, and R. d. Borst. 2017. A feedback-loop extended stress fiber growth model with focal adhesion formation. *Int. J. Solid Struct.* 128:160–173.
42. Deshpande, V. S., R. M. McMeeking, and A. G. Evans. 2006. A bio-chemo-mechanical model for cell contractility. *Proc. Natl. Acad. Sci. USA.* 103:14015–14020.
43. Keshavanarayana, P., M. Ruess, and R. de Borst. 2018. On the monolithic and staggered solution of cell contractility and focal adhesion growth. *Int. j. numer. method. biomed. eng.* 34:31388–e3220.
44. Gasser, T. C., R. W. Ogden, and G. A. Holzapfel. 2006. Hyperelastic modelling of arterial layers with distributed collagen fibre orientations. *J. R. Soc. Interface.* 3:15–35.
45. Barcelona-Estaje, E., M. J. Dalby, …, M. Salmeron-Sanchez. 2021. You Talking to Me? Cadherin and Integrin Crosstalk in Biomaterial Design. *Adv. Healthcare Mater.* 10:e2002048.
46. Ghim, M., Y. Mohamied, and P. D. Weinberg. 2021. The Role of Tricellular Junctions in the Transport of Macromolecules Across Endothelium. *Cardiovasc. Eng. Technol.* 12:101–113.
47. Srinivasan, B., A. R. Kolli, …, J. J. Hickman. 2015. TEER measuments in cells. *J. Lab. Autom.* 20:107–126.
48. Brangwynne, C. P., G. H. Koenderink, …, D. A. Weitz. 2008. Cytoplasmic diffusion: Molecular motors mix it up. *J. Cell Biol.* 183:583–587.
49. Murrell, M., P. W. Oakes, …, M. L. Gardel. 2015. Forcing cells into shape: The mechanics of actomyosin contractility. *Nat. Rev. Mol. Cell Biol.* 16:486–498.
50. Tambe, D. T., C. C. Hardin, …, X. Trepat. 2011. Collective cell guidance by cooperative intercellular forces. *Nat. Mater.* 10:469–475.
51. Chai, C. K., L. Speelman, …, F. P. T. Baaijens. 2014. Compressive mechanical properties of atherosclerotic plaques - Indentation test to characterise the local anisotropic behaviour. *J. Biomech.* 47:784–792.
52. Ebenstein, D. M., D. Coughlin, …, L. A. Pruitt. 2009. Nanomechanical properties of calcification, fibrous tissue, and hematoma from atherosclerotic plaques. *J. Biomed. Mater. Res.* 91:1028–1037.
53. Krishnan, R., D. D. Klumpers, …, G. P. Van Nieuw Amerongen. 2011. Substrate stiffening promotes endothelial monolayer disruption through enhanced physical forces. *Am. J. Physiol. Cell Physiol.* 300:146–154.
54. Vanderburgh, J. A., H. Hotchkiss, …, C. A. Reinhart-King. 2018. Substrate stiffness heterogeneities disrupt endothelial barrier integrity in a micropillar model of heterogeneous vascular stiffening. *Integr. Biol.* 10:734–746.
55. An, S. S., J. Kim, …, S. Biswal. 2009. Cell stiffness, contractile stress and the role of extracellular matrix. *Biochem. Biophys. Res. Commun.* 382:697–703.
56. van Kelle, M. A. J., P. J. A. Oomen, …, C. V. C. Bouten. 2018. Initial scaffold thickness affects the emergence of a geometrical and mechanical equilibrium in engineered cardiovascular tissues. *J. R. Soc. Interface.* 15, 20180359.
57. Tracy, E., G. Rowe, and A. J. LeBlanc. 2020. Cardiac tissue remodeling in healthy aging: The road to pathology. *Am. J. Physiol. Cell Physiol.* 319:C166–C182, –C182.
58. Chien, S. 2003. Molecular and mechanical bases of focal lipid accumulation in arterial wall. *Prog. Biophys. Mol. Biol.* 83:131–151.
59. Warboys, C. M., N. Amini, …, P. C. Evans. 2011. The role of blood flow in determining the sites of atherosclerotic plaques. *F1000 Med. Rep.* 3:5–8.
60. Chiu, J. J., and S. Chien. 2011. Effects of disturbed flow on vascular endothelium: Pathophysiological basis and clinical perspectives. *Physiol. Rev.* 91:327–387.
61. Chien, S. 2008. Effects of disturbed flow on endothelial cells. *Ann. Biomed. Eng.* 36:554–562.
62. Rostam-Alilou, A. A., H. R. Jarrah, …, M. Bodaghi. 2022. Fluid–structure interaction (FSI) simulation for studying the impact of atherosclerosis on hemodynamics, arterial tissue remodeling, and initiation risk of intracranial aneurysms. *Biomech. Model. Mechanobiol.* 21:1393–1406.
63. Paddillaya, N., S. Mahulkar, …, N. Gundiah. 2023. Controlled disturbed flows on the endothelium result in increased inflammation, nuclear lamin disruption and heterochromatin condensation. Preprint at bioRxiv. https://doi.org/10.1101/2023.08.27.555040.
64. Selman, M., and A. Pardo. 2021. Fibroageing: An ageing pathological feature driven by dysregulated extracellular matrix-cell mechanobiology. *Ageing Res. Rev.* 70:101393.
65. DePaola, N., M. A. Gimbrone, …, C. F. Dewey. 1992. Vascular endothelium responds to fluid shear stress gradients. *Arterioscler. Thromb.* 12:1254–1257.
66. Sofias, A. M., F. De Lorenzi, …, T. Lammers. 2021. Therapeutic and diagnostic targeting of fibrosis in metabolic, proliferative and viral disorders. *Adv. Drug Deliv. Rev.* 175:113831–113883.
67. Wang, Y., R. K. Kankala, …, Z. Yang. 2022. Advances in hydrogel-based vascularized tissues for tissue repair and drug screening. *Bioact. Mater.* 9:198–220.
68. Hooglugt, A., O. Klatt, and S. Huveneers. 2022. Vascular stiffening and endothelial dysfunction in atherosclerosis. *Curr. Opin. Lipidol.* 33:353–363.
69. Li, J., Y. Fang, and D. Wu. 2021. Mechanical forces and metabolic changes cooperate to drive cellular memory and endothelial phenotypes. *Curr. Top. Membr.* 87:199–253.
70. Curry, F. E., and R. H. Adamson. 2012. Endothelial glycocalyx: Permeability barrier and mechanosensor. *Ann. Biomed. Eng.* 40:828–839.
71. Dull, R. O., and R. G. Hahn. 2022. The glycocalyx as a permeability barrier: basic science and clinical evidence. *Crit. Care.* 26:273–278.

Biophysical Journal, Volume *123*

Supplemental information

A mechanical modeling framework to study endothelial permeability

Pradeep Keshavanarayana and Fabian Spill

S1 Source files

Link to download all the source files:
https://github.com/bkprdp/VE-Cadherin-Mechanical-Model-Abaqus
File descriptions :

1. `Cell_UMAT_Small_Strain.f`: User-defined material. Active and Passive stress growth, using small strain formulation.
2. `UAMP` : Random pressure amplitude
3. `Planar_Monolayer_Without_ECM.inp` : inp file for planar monolayer without ECM
4. `Planar_Monolayer_With_ECM.inp` : inp file for planar monolayer with ECM
5. `Cylindrical_Monolayer_Without_ECM.inp` : inp file for cylindrical monolayer without ECM
6. `Cylindrical_Monolayer_With_Rigid_ECM.inp` : inp file for cylindrical monolayer with fixed radial constraint representing rigid ECM
7. `Nodes_Bi_Tri_Cellular_Jncs.py` : Python script to classify nodes belonging to bi-cellular and tri-cellular junctions
8. `Planar_Monolayer_COPEN_Analysis.py` : Python script to perform quantitative analysis of the csv file containing copen values

Animations of the simulations are added as supplemental material.

S2 Traction-Separation law

In this article, traction-separation law is hypothesised to be the mechanical equivalent of the catch-slip bond law. The parameters of traction-separation law could be varied to modify the association and dissociation behaviour of VE-cadherin bonds. As explained in the main text, only three parameters are needed :

1. Stiffness of VE-cadherin bond
2. Stretch at which the VE-cadherin bond starts to dissociate
3. Stretch at which the VE-cadherin bond is completely dissociated

It can be seen in Figure S1, as the normalised stiffness is increased from 1 to 2, the maximum contact force that the bond can take is also doubled. When the maximum separation that the bond can handle before complete dissociation is increased, the time the bond takes to dissociate also increases. It can be easily seen that by changing δ_i^0, stiffness can be changed as well. Thus, by varying these parameters, quantitative comparison with experiments is possible.

S3 Cauchy stress tensor derivation

Any unit vector $\mathbf{m}$ along the fibre direction (ω,ϕ), Fig. S2, can be written as

$$\mathbf{m} = sin(\omega)cos(\phi)\mathbf{x_1} + sin(\omega)sin(\phi)\mathbf{x_2} + cos(\omega)\mathbf{x_3} \tag{1}$$

Following [1], we can write the components of active Cauchy stress tensor as

$$\sigma_{ij}^a = \frac{3}{4\pi}\int_0^{2\pi}\int_0^{\pi} \sigma^a(\omega,\phi)m_i m_j sin(\omega)d\omega d\phi \tag{2}$$

where i,j = 1,2,3. In the case of 2D geometry,

$$\sigma_{ij}^a = \frac{1}{\pi}\int_{-\pi/2}^{\pi/2} \sigma^a(\phi)m_i m_j d\phi \tag{3}$$

where i,j=1,2.

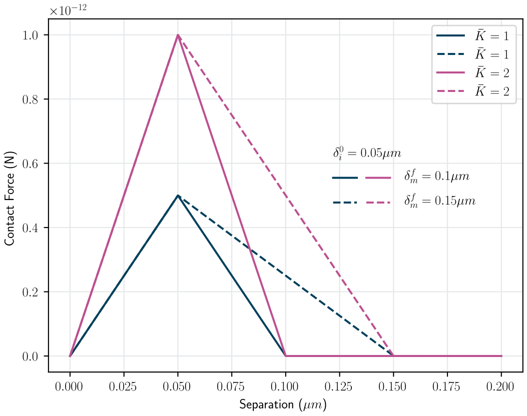

Figure S1: Parameters involved in traction separation law can be varied to study the maximum force, time of association and dissociation of VE-cadherin bonds. These parameters could also be used for simulating the differences in cell-cell and cell-ECM bonds.

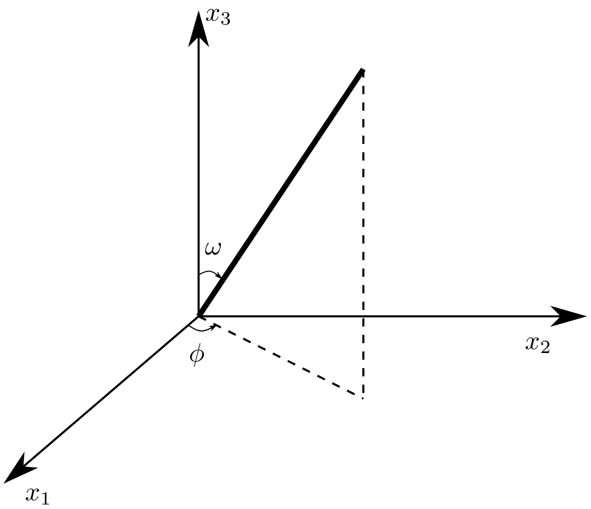

Figure S2: Orientation of fibre with respect to x_1, x_2, and x_3 axes. ω is the angle between the fibre and x_3 axis, while ϕ is the angle between the projection of fibre on x_1-x_2 plane and the x_1 axis.

S4 Finite Strain formulation

In this article, all the simulations presented used small strain formulation. This can be further extended to finite strain formulation using non-linear hyper-elastic materials. Here an example with Neo-Hookean material is given. Passive stress can thus be formulated with the strain energy function

$$\psi(I_1, J) = C_{10}(I_1 - 3) + \frac{1}{D_1}(J-1)^2 \tag{4}$$

where ψ is the strain energy density function, I_1 is the first invariant of the left Cauchy-Green tensor $\mathbf{B}$, J is the determinant of the deformation gradient $\mathbf{F}$. C_{10} and D_1 are the material parameters related to Young's modulus and Poisson ratio via Bulk modulus (K_0) and shear modulus (μ_0) as given in Eq. (5).

$$\begin{aligned} C_{10} &= \frac{\mu_0}{2} \\ D_1 &= \frac{2}{K_0} \end{aligned} \tag{5}$$

Left Cauchy-Green tensor is defined as

$$\mathbf{B} = \mathbf{F}.\mathbf{F}^{\mathbf{T}} \tag{6}$$

The constitutive equation is given by the first derivative of the strain energy function

$$\sigma^p_{ij} = \frac{2}{J}C_{10}\left(\bar{B}_{ij} - \frac{1}{3}\delta_{ij}\bar{B}_{kk}\right) + \frac{2}{D_1}(J-1)\delta_{ij} \tag{7}$$

where δ_{ij} is the Kronecker delta, and $i, j = 1, 2, 3$. $\bar{\mathbf{B}}$ is the volumetric strain tensor given by

$$\bar{\mathbf{B}} = J^{\left(\frac{-2}{3}\right)}\mathbf{B} \tag{8}$$

In general, the Jacobian matrix $\mathbf{C}$ needed for the Newton scheme used by ABAQUS, can be obtained from the equilibrium equation

$$\delta\boldsymbol{\tau} = J\mathbf{C} : \delta\mathbf{D} \tag{9}$$

where $\boldsymbol{\tau}$ is the Kirchoff stress, related to the Cauchy stress as $J\boldsymbol{\sigma}$. The rate of deformation $\mathbf{D}$ is defined as

$$\delta\mathbf{D} = \mathbf{sym}\left(\delta\mathbf{F}.\mathbf{F^{-1}}\right) \tag{10}$$

Solution of Eq. 9 can be obtained numerically by perturbing the individual component of deformation gradient $\mathbf{F}$ by a small amount ϵ as described in [2, 3]. Derivation leads to

$$\mathbf{C} = \frac{1}{J\varepsilon}\left[\boldsymbol{\tau}\left(\hat{\mathbf{F}}\right) - \boldsymbol{\tau}\left(\mathbf{F}\right)\right] \tag{11}$$

where $\hat{\mathbf{F}}$ is the perturbed deformation gradient given as

$$\hat{F}_{ij} = F_{ij} + \Delta F_{ij} \tag{12}$$

In addition, in the case of finite strain formulation, the rotation of fibres has to be taken into account during the evaluation of the active stress tensor. Thus (2) becomes

$$\sigma^a_{ij} = \frac{3}{4\pi}\int_0^{2\pi}\int_0^{\pi}\sigma^a(\omega,\phi)m^*_i m^*_j sin(\omega)d\omega d\phi \tag{13}$$

where m^*_i, m^*_j are the vectors in the current configuration evaluated as

$$m^*_i = \mathbf{R}m_i \tag{14}$$

where $\mathbf{R}$ is the rotation matrix evaluated via the decomposition of the deformation gradient as

$$\mathbf{F} = \mathbf{RU} \tag{15}$$

S5 Parameter values

Parameter values used in this article are given in Tab. S1. Some of the parameter values are obtained directly from the literature while some of them are modified to obtain qualitatively comparable results with the experiments.

Parameters	Value	Units	Reference
Passive stiffness	10	kPa	[4]
Poisson ratio	0.45	-	-
Max stress in stress fibre	1	MPa	[5]
Strain rate coefficient	0.1	s	[5]
k_f	0.01	/s	[5]
k_b	0.1	/s	[5]
Bond stiffness	1	N/m	[6]
Damage initiation	0.05	μm	[6]
Damage termination	0.15	μm	[6]
Cadherin Concentration	1e7	cadherins/m^2	-

Table S1: Baseline parameter values

S6 Convergence study

Mesh convergence analysis is performed. It can be seen in Fig. S3 that the error (evaluated wrt to results from a very fine mesh simulation) with a mesh size of 1μm is 5%. We assume that this error is acceptable for the qualitative nature of this article. Hence all the analyses performed in this article were performed with the mesh with an element size of 1μm.

S7 Effect of $\hat{C}$ on permeability

As discussed in the article, active stress, evaluated based on the calcium concentration $\hat{C}$ has a direct influence on the permeability. When $\hat{C}$ increases, the active stress in the cytoplasm increases resulting in increased permeability as shown in Fig. S4. Since an increase in cytoplasmic calcium concentration further increases the tension on cell-cell junctions, as explained in the article, intercellular random pressure load factor $\hat{\zeta}_p$ is assumed to increase with $\hat{C}$. This relation is assumed to behave as shown in Fig. S5. Further, we also show how the permeability varies with time, Fig. S7. We also saw that when ζ_p increases along with $\hat{C}$, the increase in permeability is higher than the case where ζ_p is held constant (Fig. S4). Hence, in all the simulations presented in the article, we vary ζ_p along with $\hat{C}$ following Fig. S5. The relation between ECM stiffness E and $\hat{C}$ used in this article is shown in Fig. S6.

S8 Uniform vs Random gap opening

A comparison of gap opening, when the contraction is uniform and random, is shown in Fig. S8. The simulated animations of the deformation of the endothelial monolayer, in case of uniform and random loading, can be found in the link provided in supplemental section S1

S9 Disturbed flow vs uniform flow

We define permeability as the ratio of the number of open junctions to the total number of junctions in the monolayer. As expected, when the shear stress gradient between cells increases, the probability of cell-cell junctions being open increases. In this regard, we saw that in disturbed flow conditions, where the flow is randomly varying in both x and y directions, permeability was slightly higher than that of a uniform flow condition, where all cells experience the same flow, as shown in Fig. S9. It is to be noted that the current analysis shows that the difference between uniform and disturbed flow is very small. This might be either due to the values of parameters chosen in this study or due to the lack of specific mechano-sensory channels that can sense flow such as glycocalyx. Thus, in future, this analysis will be coupled with glycocalyx which is found to play an important role in regulating permeability as well [7]. Further development could help us understand the role of flow in diseases such as atherosclerosis and other permeability-related diseases.

S10 Thickness of cell

In the article, we consider the thickness of the cell as 0.1μm. However, in literature, it has been found that thickness varies to 10 μm. In this regard, we varied the thickness of the cell and studied its effect on permeability. As the thickness of the cell is increased, keeping the cadherin concentration constant, the net force that cell-cell contact can resist without damage increases. But simultaneously, the contractile force and force due to random pressure loading on the boundary also increase. Hence the contributions balance out and we do not observe any difference in permeability due to the changing thickness of the cell as can be seen in Fig. S10. In future, this study could be extended by considering the effect of the nucleus, which will alter cell stiffness locally.

S11 Size of monolayer

We study the effect of the number of cells in the monolayer on permeability. We found that with a low number of cells in the monolayer, the variations in the magnitude of permeability at tri-cellular junctions were very high compared to that of the monolayer with 42 cells, as seen in Fig. S11b, Fig. S11c. This is due to the increased number of tri-cellular junctions used for evaluating the average permeability. In addition, as the number of cells increased, the difference in permeability between the endothelial monolayers also decreased, as shown in Fig. S11d. So, we have used a monolayer with 32 cells in all the simulations.

S12 Pseudocode

The pseudocode describing the methodology followed in the analysis presented in the article is given below.

```
Define geometry and material properties
Initial conditions (u = 0, ϵ = 0, ϵ̇ = 0, η = 0, Σ = 0)
Contact definitions
while t ≤ t_end do                                              ▷ For each time step
    while N ≤ N_ele do                                          ▷ For each element
        function EVALUATE TOTAL STRESS(u,ϵ,Σ)
            EVALUATEACTIVESTRESS(u,ϵ̇,η,σ^a)
            EVALUATEPASSIVESTRESS(u,ϵ,σ^p)
            return Σ ← σ^a + σ^p
        end function
        function EVALUATE LOAD AND BOUNDARY CONDITIONS
            if ECM is considered then
                Evaluate Cell-ECM contact
                Evaluate Cell-Cell contact
            else if ECM is not considered then
                Evaluate Cell-Cell contact
            end if
            APPLY INTERCELLULAR RANDOM PRESSURE                 ▷ Applied at cell-cell interface
            if True then
                APPLY INTERCELLULAR SHEAR STRESS                ▷ Applied at cell-cell interface
            end if
            if True then
                APPLY TRACTION DUE TO DISTURBED FLOW            ▷ Applied on top of cells
            end if
        end function
    end while
    STATICANALYSIS(Termination Criteria)
    Update (u, ϵ, ϵ̇, η)
end while
POST PROCESSING TO EVALUATE PERMEABILITY
```

S13 Simulating a specific experiment

The modelling framework presented in this article is a general computational methodology where the principles of contact mechanics have been used to study endothelial permeability. The model can be applied successfully to study a wide range of experiments either by changing the material parameters or the relation between the variables present in the model. The data-driven modifications to the model could give deeper insights into the specificities of the experiment at hand. The changes that could be made to the model with the help of data obtained from the experiments are

1. Geometry of the cell and the monolayer
2. Material parameters of the cell and the substrate
3. Material parameters for cell-cell junctions and cell-substrate junction
4. Relation between calcium concentration and intercellular pressure
5. Relation between ECM stiffness and calcium concentration
6. Distribution of random forcing functions
7. Exact distribution of force due to disturbed flow on endothelial cells evaluated using FSI or other experimental methods.

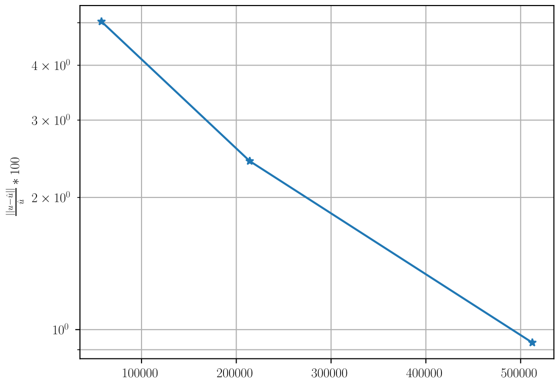

Figure S3: Mesh convergence study. Error for mesh size of 1μm is less than 5%. Hence all simulations use a mesh size of 1μm.

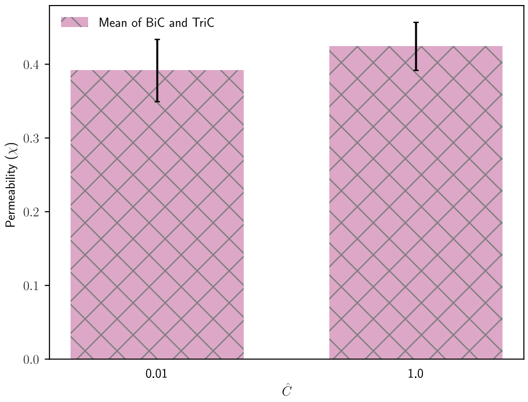

Figure S4: Increase in permeability due to increase in cytoplasmic calcium concentration keeping the intercellular pressure load factor constant. ($\hat{\zeta}_p = 0.01$ MPa). The increase is only due to an increase in active stress.

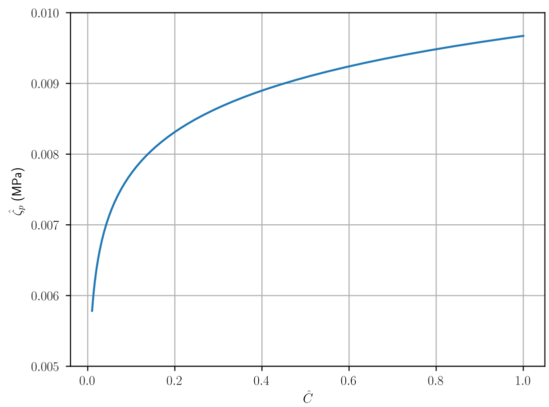

Figure S5: Relation between intercellular random pressure load factor $\hat{\zeta}_p$ and cytoplasmic calcium concentration $\hat{C}$.

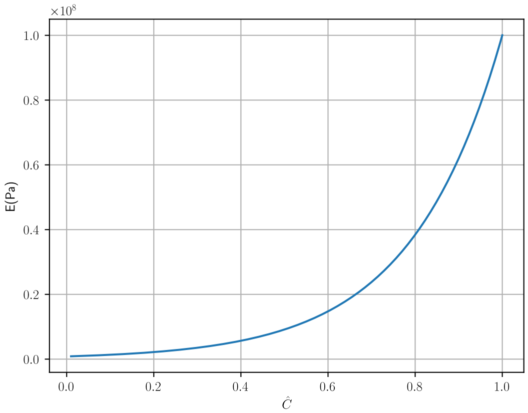

Figure S6: Relation between ECM stiffness and cytoplasmic calcium concentration $\hat{C}$.

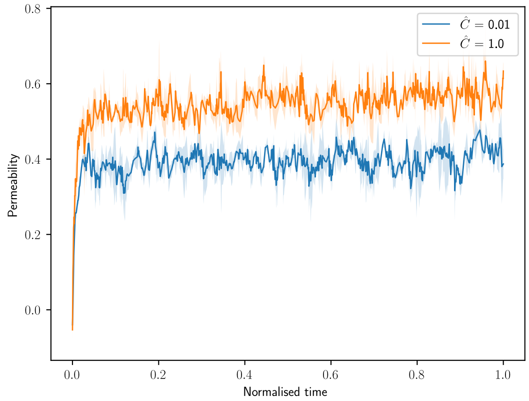

Figure S7: Variation of permeability over time. With an increase in $\hat{C}$, permeability increases.

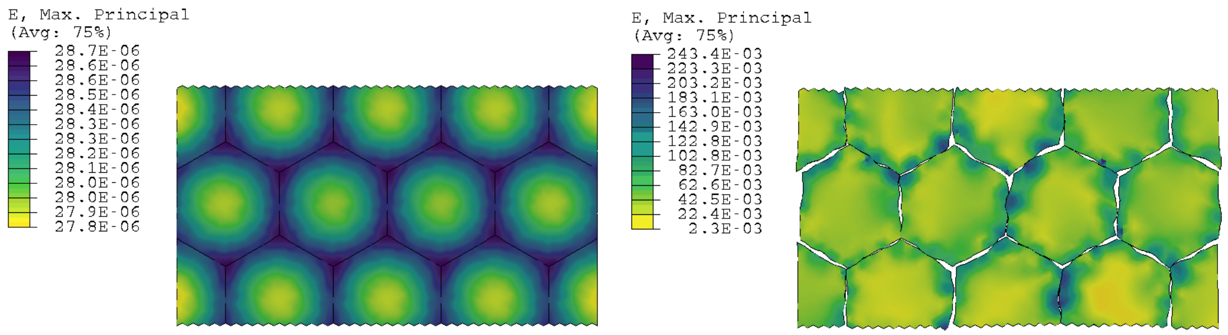

(a) **(b)**

Figure S8: Strain field in a planar monolayer subjected to different types of loading. a) Due to the uniform contraction of cells, strain is high at a tri-cellular junction compared to the bi-cellular junction. b) Due to random loads between cells, strain is high either at a bi-cellular or tri-cellular junction depending on the load. Thus, the location of high permeability depends on the strain distribution in the endothelial monolayer.

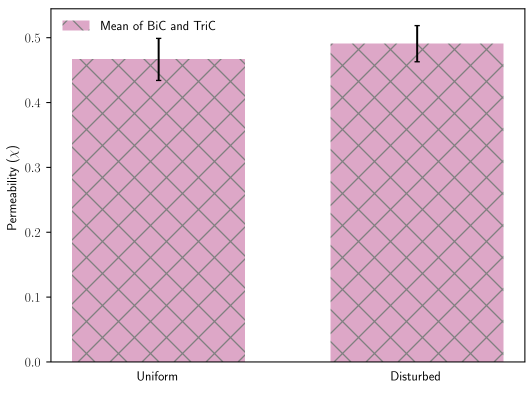

Figure S9: Variation in permeability due to uniform and disturbed flows. The difference in permeability is not high. This indicates the need for the inclusion of suitable mechano-sensors such as glycocalyx in the model.

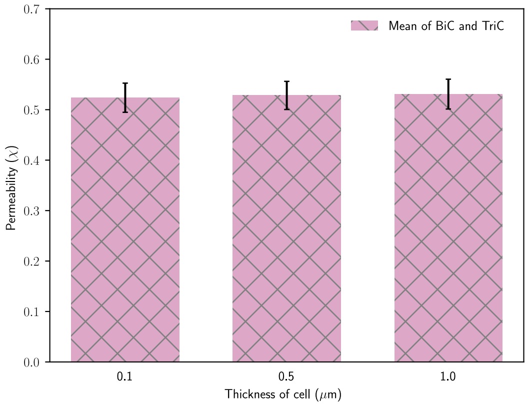

Figure S10: Variation in permeability due to change in thickness of the cell. The difference in permeability due to changing thickness is not significant.

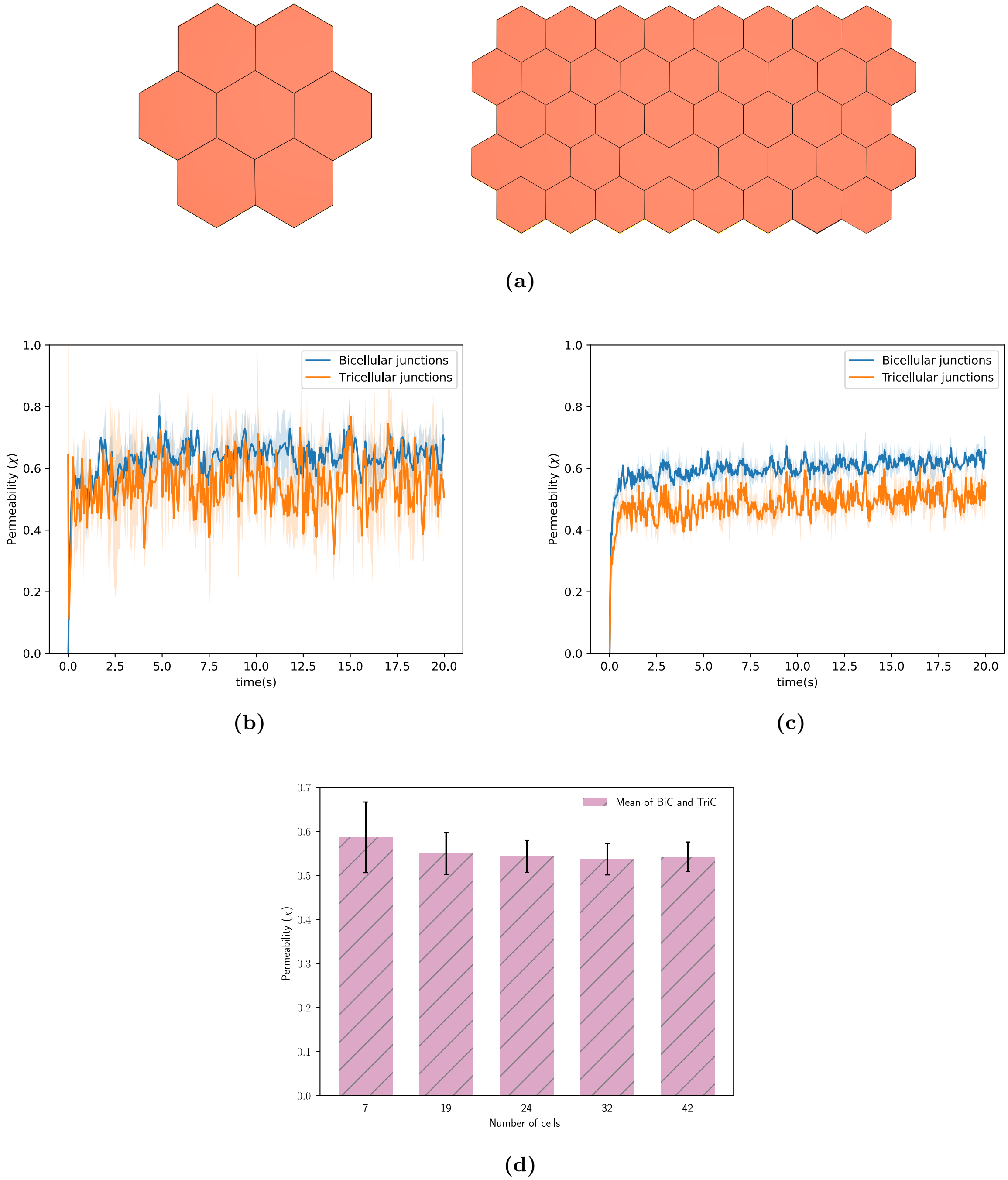

Figure S11: Variation in permeability over time for planar monolayer with differing number of cells. a) Schematic of planar monolayer with 7 and 42 cells. b) The variation in permeability at tri-cellular junctions in the case of the monolayer with 7 cells is very high. This is because there are only 6 tricellular junctions available over which the average is evaluated. c) Upon an increase in the number of tri-cellular junctions by increasing the number of cells to 42, the behaviour is smoothened. d) Upon an increase in the number of cells in the monolayer from 7 to 19, we observe a drop in the permeability. However, upon increasing the number of cells further, permeability does not vary much. We use 32 cells in all the simulations using the planar endothelial monolayer in this article.

Bibliography

[1] V. S. Deshpande, R. M. McMeeking, and A. G. Evans, "A model for the contractility of the cytoskeleton including the effects of stress-fibre formation and dissociation," *Proc. R. Soc. A Math. Phys. Eng. Sci.*, vol. 463, no. 2079, pp. 787–815, 2007.

[2] C. Miehe, "Numerical computation of algorithmic (consistent) tangent moduli in large-strain computational inelasticity," *Comput. Methods Appl. Mech. Eng.*, vol. 134, no. 3-4, pp. 223–240, 1996.

[3] Wei Sun, Elliot L. Chaikof, and M. E. Levenston, "Numerical Approximation of Tangent Moduli for Finite Element Implementations of Nonlinear Hyperelastic Material Models," *J Biomech Eng.*, vol. 130, no. 6, 2008.

[4] H. Sato, M. Katano, T. Takigawa, and T. Masuda, "Estimation for the elasticity of vascular endothelial cells on the basis of atomic force microscopy and Young's modulus of gelatin gels," *Polym. Bull.*, vol. 47, no. 3-4, pp. 375–381, 2001.

[5] P. Keshavanarayana, M. Ruess, and R. de Borst, "A feedback-loop extended stress fiber growth model with focal adhesion formation," *Int. J. Solids Struct.*, vol. 128, pp. 160–173, 2017.

[6] P. Panorchan, J. P. George, and D. Wirtz, "Probing Intercellular Interactions between Vascular Endothelial Cadherin Pairs at Single-molecule Resolution and in Living Cells," *J. Mol. Biol.*, vol. 358, no. 3, pp. 665–674, 2006.

[7] R. O. Dull and R. G. Hahn, "The glycocalyx as a permeability barrier: basic science and clinical evidence," *Crit. Care*, vol. 26, no. 1, pp. 1–8, 2022.
